# DNA methylation profiling in peripheral lung tissues of smokers and patients with COPD

**DOI:** 10.1186/s13148-017-0335-5

**Published:** 2017-04-14

**Authors:** Isaac K. Sundar, Qiangzong Yin, Brian S. Baier, Li Yan, Witold Mazur, Dongmei Li, Martha Susiarjo, Irfan Rahman

**Affiliations:** 1 Department of Environmental Medicine, University of Rochester Medical Center, Box 850, 601 Elmwood Avenue, Rochester, 14642 NY USA; 2 Department of Clinical & Translational Research, University of Rochester Medical Center, Rochester, NY USA; 3 Department of Biostatistics and Bioinformatics, Roswell Park Cancer Institute, Buffalo, NY USA; 4 Heart and Lung Center, University of Helsinki and Helsinki University Hospital, Helsinki, Finland

**Keywords:** DNA methylation, Lung, Epigenetics, COPD, Smokers, Pyrosequencing

## Abstract

**Background:**

Epigenetics changes have been shown to be affected by cigarette smoking. Cigarette smoke (CS)-mediated DNA methylation can potentially affect several cellular and pathophysiological processes, acute exacerbations, and comorbidity in the lungs of patients with chronic obstructive pulmonary disease (COPD). We sought to determine whether genome-wide lung DNA methylation profiles of smokers and patients with COPD were significantly different from non-smokers. We isolated DNA from parenchymal lung tissues of patients including eight lifelong non-smokers, eight current smokers, and eight patients with COPD and analyzed the samples using Illumina’s Infinium HumanMethylation450 BeadChip.

**Results:**

Our data revealed that the differentially methylated genes were related to top canonical pathways (e.g., G beta gamma signaling, mechanisms of cancer, and nNOS signaling in neurons), disease and disorders (organismal injury and abnormalities, cancer, and respiratory disease), and molecular and cellular functions (cell death and survival, cellular assembly and organization, cellular function and maintenance) in patients with COPD. The genome-wide DNA methylation analysis identified suggestive genes, such as *NOS1AP*, *TNFAIP2*, *BID*, *GABRB1*, *ATXN7*, and *THOC7* with DNA methylation changes in COPD lung tissues that were further validated by pyrosequencing. Pyrosequencing validation confirmed hyper-methylation in smokers and patients with COPD as compared to non-smokers. However, we did not detect significant differences in DNA methylation for *TNFAIP2*, *ATXN7*, and *THOC7* genes in smokers and COPD groups despite the changes observed in the genome-wide analysis.

**Conclusions:**

Our study suggests that DNA methylation in suggestive genes, such as *NOS1AP*, *BID*, and *GABRB1* may be used as epigenetic signatures in smokers and patients with COPD if the same is validated in a larger cohort. Future studies are required to correlate DNA methylation status with transcriptomics of selective genes identified in this study and elucidate their role and involvement in the progression of COPD and its exacerbations.

**Electronic supplementary material:**

The online version of this article (doi:10.1186/s13148-017-0335-5) contains supplementary material, which is available to authorized users.

## Background

Cigarette smoking is the main etiological factor in the pathogenesis of chronic obstructive pulmonary disease (COPD). Several mechanisms have been proposed in the pathogenesis of COPD, such as oxidative stress, inflammation, protease/antiprotease, epigenetics, apoptosis, and cellular senescence [1]. Cigarette smoke (CS) is known to affect the transcriptional regulation of upstream and downstream target genes involved in different canonical pathways that are implicated in the progression of COPD. However, not all the alterations in the pathogenesis of COPD, e.g., steroid resistance, acute exacerbations, and comorbidity, can be explained by transcriptional changes or cellular abnormalities alone [2, 3].

Epigenetic mechanisms, specifically methylation status of the DNA at specific CpG sites, are known to play a crucial role in several chronic inflammatory diseases including cancer and aging [2, 3]. DNA methylation plays an important role in transcriptional regulation, e.g., gene silencing [1, 2]. Environmental and genetic factors, such as CS exposure, diet, genetic variation, and aging, trigger oxidative stress that can affect the promoter CpG methylation by recruiting methyl CpG binding protein 2 and DNA methyltransferases onto various promoters [1, 2]. Alteration in the methylation status of the promoter affects expression of tumor suppressor, oncogenes, and pro- and anti-inflammatory genes [2]. Previously, studies were conducted using sputum, whole blood, peripheral blood leukocytes, WBCs, alveolar macrophages, small airway epithelium, lung tissues, including buccal brushings, and bronchial brushings of small airways to investigate DNA methylation sites associated with smokers and patients with COPD [4–15]. Different methods of DNA methylation profiling were conducted to assess the epigenetic disruption in key genes and canonical pathways associated with smoking history and COPD status [5–15]. However, the DNA methylation profiling in parenchymal lung tissues of smokers and patients with COPD and their relationship between gene-specific DNA methylation, smoking, and COPD disease progression remains unclear.

DNA methylation is a reversible gene regulatory modification which is shown to be altered by tobacco smoke. Current smokers with a gene-specific DNA hyper- or hypo-methylation can be highly susceptible to disease development based on the patterns of genomic DNA methylation profiles. A growing body of evidence suggests that DNA methylation status in gene promoters can be used as a novel epigenetic biomarker for smoking-related chronic lung diseases, such as COPD and its exacerbations as well as lung cancer [10, 16–19]. The effects of differential DNA methylation patterns mediated by tobacco smoke, particularly in lung tissues from healthy lifelong non-smokers in comparison with smokers and patients with COPD, remains unclear. In the present study, we used a genome-wide DNA methylation analysis (Illumina’s Infinium 450K BeadChip array) combined with pyrosequencing approaches to identify novel suggestive genes as epigenetic signatures of DNA methylation in lung tissues. This study could offer promise as measures of tobacco smoke exposure or toxicity and ultimately serve as an indicator for the susceptibility, progression, pathogenesis, and exacerbations of smoking-related chronic lung diseases. Some of the results have been reported in the form of an abstract [20].

## Methods

### Ethics statement and scientific rigor/reproducibility

The lung tissue specimens from normal, lifelong non-smokers, smokers, and patients with COPD were collected by the Department of Medicine and Pathology, Helsinki University Central Hospital. The clinical characteristics of the subjects/patients used in this study are summarized (Table 1). We used a rigorous/robust and unbiased approach throughout the experimental plans and during analyzing the data so as to ensure that our data are reproducible along with by full and detailed reporting of both methods and raw/analyzed data. All the key biological and/or chemical resources that are used in this study were validated and authenticated (methods and resources) and are of scientific standard from commercial sources. Our results adhere to NIH standards of reproducibility and scientific rigor.

**Table 1:** General clinical characteristics of non-smokers, smokers, and COPD patients

Parameters	Non-smokers	Smokers	COPD
Number of subjects	8	8	8
M:F ratio	6:2	7:1	5:3
Male:female (%)	(75%:25%)	(87.5%:12.5%)	(62.5%:37.5%)
Age, year	62.62 ± 1.87	61 ± 2.33	56.1 ± 2.57
	(55–69)	(52–71)	(45–63)
Smoking, pack years	–	27.14 ± 5.96^***^	28.8 ± 4.32^***^
		(10–50)^a^	(10–43)^a^
FEV_1_ % predicted	100.28 ± 5.55	88.75 ± 4.04	23 ± 4.41^***; ###^
	(81–124)^b^	(68–102)	(10–43)
FEV_1_/FVC %	0.79 ± 0.01	0.81 ± 0.02	0.33 ± 0.03^***; ###^
	(0.73–0.85)^b^	(0.75–0.95)	(0.25–0.5)

^a^No pack years details for one subject in the smokers and COPD groups

^b^No FEV_1_ % predicted (one subject) and FEV_1_/FVC % values (two subjects) in the non-smokers group

^***^ *P* < 0.001 vs. non-smokers, ^###^ *P* < 0.001 vs. smokers

*M:F* Male to Female ratio, *FEV*_*1*_ forced expiratory volume in 1 s

### Human lung tissues

Lung tissue specimens from 24 subjects/patients including 8 lifelong non-smokers, 8 current smokers with normal lung function, and 8 patients with COPD undergoing resection for suspected lung tumor (either malignant or nonmalignant-local carcinoma or hamartoma) or lung transplantation from the Department of Medicine and Pathology, Helsinki University Hospital as described in our previous study [21, 22]. Tumor-free peripheral lung tissues were immediately stored at −80 °C for DNA extraction. The clinical characteristics of the subjects/patients used in this study are provided (Table 1).

### Genomic DNA isolation and DNA methylation profiling

Lung tissues were collected snap frozen and stored at −80 °C. Genomic DNA was extracted from lung tissue using the Qiagen DNAeasy kit (Qiagen, Valencia, AC) according to the manufacturer’s instructions. Briefly, 1 μg genomic DNA was bisulfite-converted using EZ DNA Methylation Kit (Zymo Research, Irvine, CA). The bisulfite-converted DNA samples were assayed using Infinium HumanMethylation450 BeadChip array from Illumina (San Diego, CA). A total of 485,512 CpG sites were probed for each sample that was processed as per the manufacturer’s protocol. Image and data analysis of the BeadChips were performed using the Illumina iScan Reader. The image data is then transferred to Illumina GenomeStudio data processing, validation of assay controls, and report generation using the methylation module. The level of methylation for the interrogated locus is determined by calculating the ratio of the fluorescent signals from methylated vs. unmethylated sites. From the genome-wide DNA methylation data, we identified specific genes based on their top canonical pathways and biological functions by functional network analysis using ingenuity pathway analysis (IPA) that were significantly hyper-methylated in lung tissues of smokers and patients with COPD compared to non-smokers for validation by pyrosequencing.

### Statistical approaches for DNA methylation analysis and functional network analysis

Quality control (QC) was first performed on DNA methylation data by filtering out probes with any of the following conditions: (1) Missing methylation beta value in any of the samples, (2) probes on X and Y chromosome, (3) any probes associated with SNP, (4) any probes that cannot unambiguously mapped to the Human Reference Genome, and (5) ensured at least 75% samples with detection *P* value <0.05 in each of the three groups (Non-smokers, NS; Smokers, S; and COPD, C). After the above QC filtering, we have a total of 276,260 probes. Second, the raw DNA methylation level data (beta values) were normalized using the SWAN normalization method in minfi package [23]. The SWAN method was able to correct the bias introduced by the two types of probes in the Illumina 450K platform due to chemistry differences in those probes [24]. The quality-controlled and normalized DNA methylation data from the COPD, smoker, and non-smoker groups conducted at two different time points were then combined together. A low DNA methylation level filtering was applied to the post-preprocessed DNA methylation data to filter out DNA methylation loci with maximum methylation level below 0.15. The difference in methylation levels between different groups at each remained methylation loci was examined using a general linear model approach after adjusting the time effect in the model using the limma package in R/Bioconductor [25]. The estimates of the group differences were examined using a moderated *t* statistics with empirical Bayes approach to shrink the standard deviations for obtaining robust estimates. The top candidate genes/probes were selected based on uncorrected *P* values since none of the CpG sites were significant after adjusting the raw *P* values using the Benjamini-Hochberg procedure. Although those top candidate gene/probes were selected based on uncorrected *P* values, they did indicate the observed differences between groups that may likely be true positives and possibly reach statistical significance after multiplicity adjustment given a larger sample size which will be further evaluated in future studies. Linear contrasts within the general linear models were conducted for pairwise comparisons between groups (COPD vs. smoker; COPD vs. non-smoker; smoker vs. non-smoker). Top candidates in each pairwise comparison were selected by test statistics and *P* values (with unadjusted raw *P* value *P* < 0.001) from comparisons. Venn diagrams were used to show the overlap of identified top candidates of methylated loci between different pairwise comparisons. Heatmaps of methylation levels from selected DNA methylation loci were generated to show the differences between groups.

For biological insights into top differential methylation changes in relation to smokers vs. non-smokers and COPD vs. non-smokers, we implemented a functional network analysis. Genes annotated from selected differentially methylated probes (DMPs) *P* value <0.001 were included in the analysis of gene regulation network. We used a core analysis of ingenuity pathway analysis (Ingenuity Systems, Inc., Redwood City, CA, USA). Additionally, statistical analysis of significance for genome-wide DNA methylation analysis represented as box plots and pyrosequencing data analysis included as part of the main figures and supplemental data were calculated using one-way analysis of variance (ANOVA) followed by Tukey’s multiple comparisons test using GraphPad Prism 6. The results are shown as the mean ± SEM unless otherwise indicated. *P* < 0.05 is considered as statistically significant.

### Pyrosequencing analysis

Validation of selective gene-specific CpGs identified from HumanMethylation450 BeadChip analysis was performed using the same genomic DNA used for genome-wide methylation analysis as described above. We validated the methylation of five CpG sites (*NOS1AP*, *TNFAIP2*, *BID*, *GABRB1*, *ATXN7*, and *THOC7*) identified by the 450K array mentioned above and two other CpG sites [*AHRR* (cg21161138) and *SERPINA1* (cg02181506)] based on prior studies [11, 26]. When we designed the assay for pyrosequencing of target CpG sites, we included additional CpG sites along with the CpG site identified based on genome-wide DNA methylation data for all the selected subset of genes for pyrosequencing. Forward and reverse primers are designed using the Pyromark Assay Design Software. Bisulfite conversion and cleanup of DNA samples were performed by using EpiTect Fast Bisulfite Conversion Kits (Qiagen). DNA was amplified in PCR using the PyroMark PCR kit (Qiagen). Pyrosequencing was performed using the PyroMark Q24 Advanced as per the manufacturer’s instructions. Using the PyroMark CpG Software 2.0, CpG methylation percentages were calculated based on the height of the T and C peaks at the methylation site and applying the formula (C/C + T) × 100. The forward and reverse PCR primers and sequencing primer for specific CpGs are listed (Table 8).

## Results

Genome-wide DNA methylation data analysis was performed using lung tissues from eight patients with COPD (GOLD stages: III–IV) with mean forced expiratory volume in 1 s, FEV_1_ predicted (23 ± 4.41), eight smokers with FEV_1_ predicted (88.75 ± 4.04) and eight non-smokers (controls) with normal spirometry (Table 1). There were no significant differences between non-smokers, smokers, and patients with COPD based on their sex and age. All the smokers and patients with COPD had higher pack years of cigarettes smoked compared to non-smokers (*P* < 0.001).

### Differential methylation

We hypothesized that genome-wide lung DNA methylation profiles of smokers and patients with COPD would be significantly different from non-smokers (controls). Boxplot was generated using the boxplot function in *R*. Boxplot was used to describe the distribution of pre-processed DNA methylation level (*β* values). The central 50 percentile of the *β* values range from 0.35 to 0.90. The spread of the *β* values were slightly varied within each group. No obvious differences between groups were observed for distribution of *β* values (Additional file 1: Figure S1). Differentially methylated probe (DMP) analysis revealed a total of 10 CpG sites that were possibly differentially methylated between smokers vs. non-smokers, a total of 280 CpG sites that were possibly differentially methylated between COPD vs. non-smokers, and a total of 10 CpG sites that were possibly differentially methylated between COPD vs. smokers (unadjusted raw *P* value *P* < 0.001) (Additional file 2: Table S1, Additional file 3: Table S2 and Additional file 4: Table S3). Similarly, DMP analysis revealed 115 CpG sites (smokers vs. non-smokers), 1961 CpG sites (COPD vs. non-smokers), and 136 CpG sites (COPD vs. smokers) (unadjusted raw *P* value *P* < 0.01) (Additional file 5: Table S4, Additional file 6: Table S5 and Additional file 7: Table S6). Finally, DMP analysis with unadjusted raw *P* value *P* < 0.05 revealed a total of 10,363 CpG sites in smokers vs. non-smokers, a total of 34,151 CpG sites in COPD vs. non-smokers, and a total of 11,339 CpG sites that were differentially methylated between COPD vs. smokers (Additional file 8: Table S7, Additional file 9: Table S8 and Additional file 10: Table S9).

**Fig. 1:**
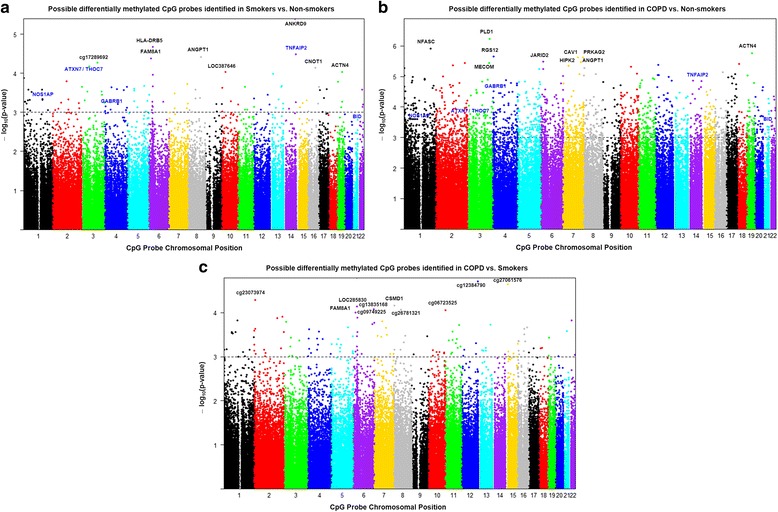
Manhattan plots showing distribution of possible differentially methylated CpG sites identified in this study across chromosomes. **a** Differential methylation analysis between smokers and non-smokers presented by chromosomal location (*x* axis). **b** Differential methylation analysis between COPD and non-smokers presented by chromosomal location (*x* axis). **c** Differential methylation analysis between COPD and smokers presented by chromosomal location (*x* axis). The *y* axis represents the negative log *P* value of their association. The genes marked in *blue* color are the once validated by pyrosequencing analysis. The *black dotted horizontal line* indicates the genome-wide significance threshold of *P* < 0.001. The top candidates in each pairwise comparison were selected by test statistics and unadjusted *P* values for comparisons (*P* < 0.001 is commonly used as a cutoff for relatively small unadjusted raw *P* value)

**Fig. 2:**
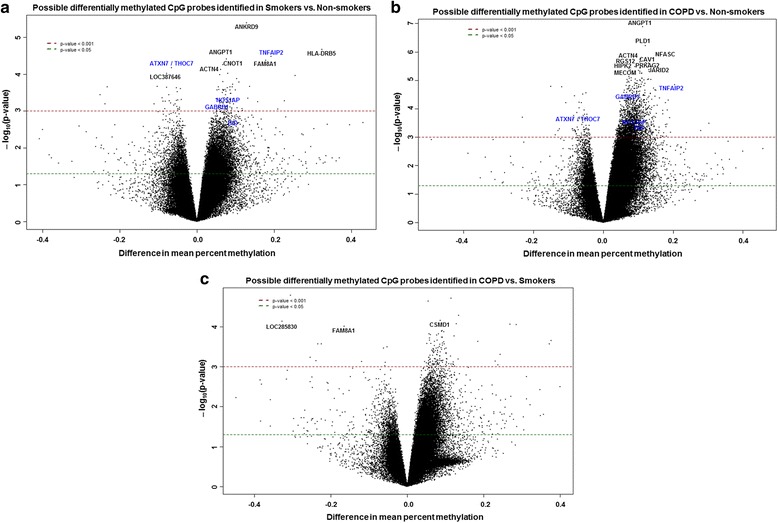
*Volcano plots* showing possible differentially methylated CpG sites identified in this study. **a** Differential methylation analysis revealed top 10 CpG sites and their genes significantly associated with smokers with *P* < 0.001. Difference in mean percent methylation represents the difference in mean methylation between smokers vs. non-smokers (control). **b** Differential methylation analysis revealed top 10 CpG sites and their genes significantly associated with COPD with *P* < 0.001. Difference in mean percent methylation represents the difference in mean methylation between COPD vs. non-smokers (control). **c** Differential methylation analysis revealed CpG sites and their genes significantly associated with COPD compared to smokers with *P* < 0.001. Difference in mean percent methylation represents the difference in mean methylation between COPD vs. smokers (control). The *y* axis represents the negative log *P* value of their association. The genes marked in *blue* color are the once validated by pyrosequencing analysis. The *red* and *green dotted horizontal lines* indicate the genome-wide significance threshold of *P* < 0.001 and *P* < 0.05, respectively. The top candidates in each pairwise comparison were selected by test statistics and unadjusted *P* values for comparisons (*P* < 0.001 is commonly used as a cutoff for relatively small unadjusted raw *P* value)

**Fig. 3:**
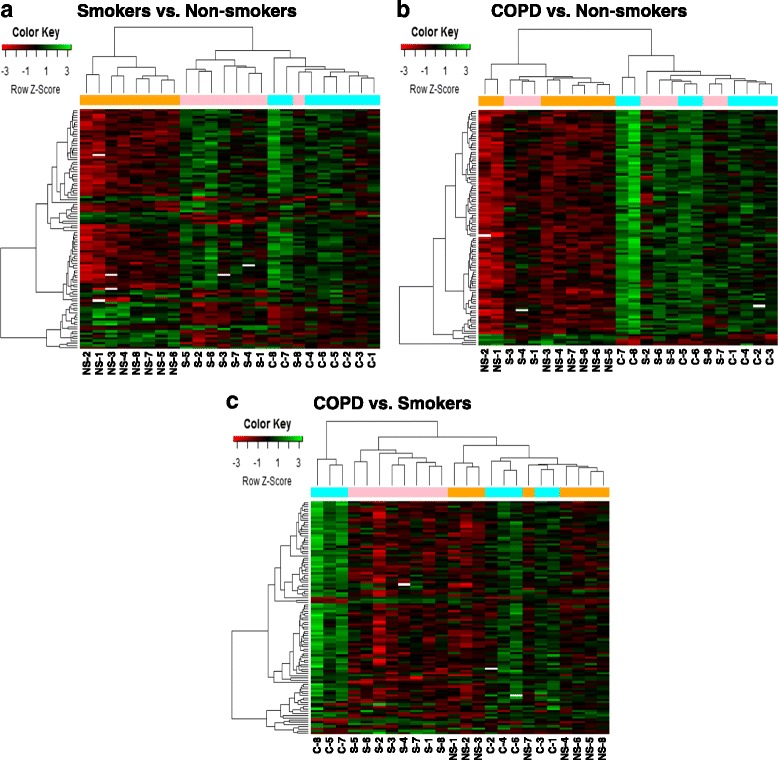
Hierarchical cluster analysis of differentially methylated CpG sites identified in this study. **a** Heatmap of top 100 differentially methylated CpG sites in smokers vs. non-smokers group. **b** Heatmap of top 100 differentially methylated CpG sites in COPD vs. non-smokers group. **c** Heatmap of top 100 differentially methylated CpG sites in COPD vs. smokers group. Additionally for cluster analysis, we have also included the five genes that were chosen for validation for pyrosequencing along with the top 100 genes. The *green* color in the heatmap denotes hyper-methylated loci, and the *red* color in the heatmap denotes the hypo-methylated loci. Target id and gene list for the top 100 differentially methylated probes (**a**–**c**) are included in the Additional file 11: Table S10, Additional file 12: Table S11 and Additional file 13: Table S12

Manhattan plot was generated using the Manhattan.plot function in *R*. Manhattan plot showed the negative *P* values on log10 scale on each chromosome with relatively small unadjusted raw *P* values corresponding to larger–log10 (*p* value). The chromosome-wide distribution of CpG sites and their comparison groups (non-smokers vs. smokers, non-smokers vs. COPD, and COPD vs. smokers) were shown (Fig. 1a–c and Tables 2, 3, and 4). Similarly, volcano plot was generated using the volcano plot function in limma package in *R*. Volcano plot is a scatter plot of log odds ratios vs. log fold change. The top 10 CpGs sites and associated genes including others CpG sites that were chosen for validation by pyrosequencing (*NOS1AP*, *TNFAIP2*, *BID*, *GABRB1*, *ATXN7*, and *THOC7*) based on their biological function with corresponding DNA methylation loci that were differentially methylated between different comparison groups are shown in the volcano plots (Fig. 2a–c and Tables 2, 3, and 4). Venn diagram was generated using the Venn diagram function in limma package in *R*. Venn diagram shows the overlap of DNA methylation loci that are shared between different comparisons (Additional file 1: Figure S2). The total number of overlapping genes and probes under each comparison groups shown in Venn diagram were not the same since a few of these did not annotate to genes (Additional file 2: Table S1, Additional file 3: Table S2 and Additional file 4: Table S3). Heatmaps were generated using the heatmap.2 function in the gplot package in *R*. We have included the DNA methylation loci and samples groups (NS, non-smokers; S, smokers, and C, COPD) for cluster analysis using the hierarchical clustering method. The green color in the heatmap denotes hyper-methylated loci, and the red color in the heatmap denotes the hypo-methylated loci. The sample groups are denoted by different colors. The top 100 genes along with the genes chosen for validation by pyrosequencing were included as part of the cluster analysis in smoker vs. non-smokers (Fig. 3a), COPD vs. non-smokers (Fig. 3b), and COPD vs. smokers (Fig. 3c), including the list of DMPs (Additional file 11: Table S10, Additional file 12: Table S11 and Additional file 13: Table S12).

**Table 2:** Top 10 differentially methylated CpG sites in smokers compared to non-smokers, sorted by *P* value

CpG site	Gene symbol	Chromosome	^a^Probe Type	*P* value	^b^Gene location of first annotated transcript	^c^Island location
cg14531093	*ANKRD9*	14	I	4.04E-06	Body	Island
cg15011943	*HLA-DRB5*	6	II	2.16E-05	Body	S_Shelf
cg18620571	*TNFAIP2*	14	I	3.28E-05	Body	Island
cg22837763	*ANGPT1*	8	II	3.86E-05	Body	
cg10154826	*FAM8A1*	6	II	4.26E-05	1^st^ Exon	Island
cg17289692		3	II	5.48E-05		
cg07753241	*ATXN7;THOC7*	3	II	6.74E-05	TSS1500	Island
cg01096617	*CNOT1*	16	II	7.50E-05	Body	
cg25383568	*ACTN4*	19	I	9.46E-05	Body	Island
cg20377766	*LOC387646*	10	II	9.63E-05	TSS1500	Island

^a^Probe type I or type II design specified on the assay

^b^Location relative to the first listed transcript

^c^S_ denote the downstream end of the island region

**Table 3:** Top 10 differentially methylated CpG sites in COPD compared to non-smokers, sorted by *P* value

CpG site	Gene symbol	Chromosome	^a^Probe Type	*P* value	^b^Gene location of first annotated transcript	Island location
cg05275153	*RGS12*	4	I	2.24E-06	Body	
cg06746365	*PRKAG2*	7	II	2.48E-06	Body	
cg08830492	*NFASC*	1	I	1.24E-06	5UTR	Island
cg14630106	*HIPK2*	7	II	3.35E-06	Body	
cg15558717	*CAV1*	7	II	2.40E-06	Body	
cg22837763	*ANGPT1*	8	II	1.30E-07	Body	
cg22920586	*PLD1*	3	II	5.93E-07	5UTR	
cg24408769	*JARID2*	6	I	3.30E-06	Body	
cg25010400	*MECOM*	3	II	3.64E-06	Body	
cg25383568	*ACTN4*	19	I	1.75E-06	Body	Island

^a^Probe type I or type II design specified on the assay

^b^Location relative to the first listed transcript

**Table 4:** Top 10 differentially methylated CpG sites in COPD compared to smokers, sorted by *P* value

CpG site	Gene symbol	Chromosome	^a^Probe Type	*P* value	^b^Gene location of first annotated transcript	^c^Island location
cg06723525		10	II	8.96E-05		S_Shore
cg08236285	*CSMD1*	8	II	6.95E-05	Body	
cg09749225		6	I	8.57E-05		Island
cg10154826	*FAM8A1*	6	II	9.94E-05	1^st^Exon	Island
cg12035144	*LOC285830*	6	II	7.28E-05	TSS1500	S_Shore
cg12384790		12	II	1.96E-05		N_Shelf
cg13835168		6	II	1.64E-05		
cg23073974		2	II	5.27E-05		
cg26781321		8	II	8.79E-05		
cg27061576		15	II	2.28E-05		

^a^Probe type I or type II design specified on the assay

^b^Location relative to the first listed transcript

^c^N_ and S_ denote the upstream and downstream end of the island region, respectively

**Fig. 4:**
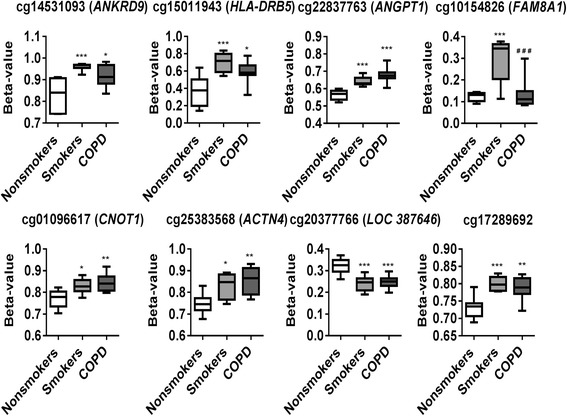
Differentially methylated CpG sites associated with smokers. Differential methylation analysis revealed CpG sites in genes significantly associated with smokers with a *P* value less than 0.05. Difference in mean beta values represents the difference in mean methylation between smokers and non-smokers (controls). The *y* axis represents the beta value. The gene symbol associated with CpG probes are provided in *parenthesis*. Data are represented as a *box plot* which displays the full range of variation (from min to max), the likely range of variation (the IQR) and a typical value (the median) for *n* = 8/group, and the significance determined using one-way ANOVA (Tukey’s multiple comparisons test). *P < 0.05, **P < 0.01, ***P < 0.001, vs. non-smokers; ^###^ *P* < 0.001 vs. smokers

**Fig. 5:**
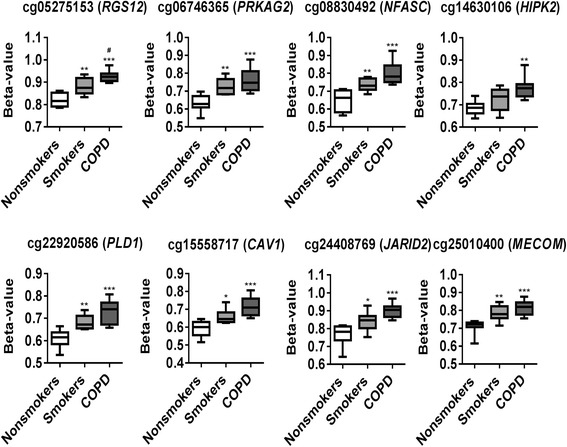
Differentially methylated CpG sites associated with COPD. Differential methylation analysis revealed CpG sites in genes significantly associated with COPD with a *P* value less than 0.05. Difference in mean beta values represents the difference in mean methylation between COPD and non-smokers (controls). The *y* axis represents the beta value. The gene symbol associated with CpG probes are provided in *parenthesis*. Data are represented as a *box plot* which displays the full range of variation (from min to max), the likely range of variation (the IQR) and a typical value (the median) for *n* = 8/group, and the significance determined using one-way ANOVA (Tukey’s multiple comparisons test). *P < 0.05, **P < 0.01, ***P < 0.001, vs. non-smokers; ^#^ *P* < 0.05 vs. smokers

**Fig. 6:**
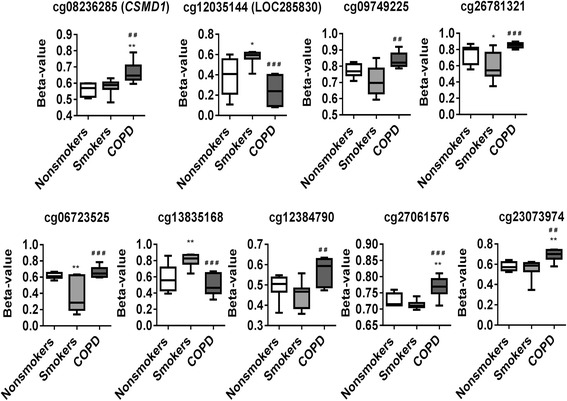
Differentially methylated CpG sites associated with COPD vs. smokers. Differential methylation analysis revealed CpG sites in genes significantly associated with smokers vs. COPD with a *P* value less than 0.05. Difference in mean beta values represents the difference in mean methylation between smokers and COPD. The *y* axis represents the beta value. The gene symbol associated with CpG probes are provided in *parenthesis*. Data are represented as a *box plot* which displays the full range of variation (from min to max), the likely range of variation (the IQR) and a typical value (the median) for *n* = 8/group, and the significance determined using one-way ANOVA (Tukey’s multiple comparisons test). *P < 0.05, **P < 0.01, vs. non-smokers; ^##^ *P* < 0.01, ^###^ *P* < 0.001 vs. smokers

From genome-wide methylation data, when we compared possible differentially methylated probes and their associated genes between smokers vs. non-smokers, results revealed the top 10 CpG sites such as *ANKRD9* (cg14531093), *HLA-DRB5* (cg15011943), *TNFAIP2* (cg18620571), *ANGPT1* (cg22837763), *FAM8A1* (cg10154826), cg17289692, *ATXN7* and *THOC7* (cg07753241), *CNOT1* (cg01096617), *ACTN4* (cg25383568), and *LOC387646* (cg20377766) (Fig. 4 and Table 2). Similarly, when we compared possible differentially methylated probes and their associated genes between COPD vs. non-smokers, results revealed the top 10 CpG sites such as *ANGPT1* (cg22837763), *PLD1* (cg22920586), *NFASC* (cg08830492), *ACTN4* (cg25383568), RGS12 (cg05275153), *CAV1* (cg15558717), *PRKAG2* (cg06746365), *JARID2* (cg24408769), *HIPK2* (cg14630106), and *MECOM* (cg25010400) (Fig. 5 and Table 3). Finally, when we compared possible differentially methylated probes and their associated genes between COPD vs. smokers, results revealed of the top 10 CpG sites 3 were associated with genes CSMD1 (cg08236285), LOC285830 (cg12035144), and *FAM8A1* (cg10154826). The remaining seven CpG sites did not annotate to genes (cg13835168, cg12384790, cg27061576, cg23073974, cg0749225, cg26781321, and cg06723525) (Fig. 6 and Table 4). To further examine the differential methylation results associated with smokers vs. non-smokers and COPD vs. non-smokers, pathway analyses were performed using IPA. The possible differentially methylated genes identified with unadjusted raw *P* value *P* < 0.001 revealed significant enrichment of genes related to top diseases and biological functions (Tables 5 and 6) including enriched canonical pathways (Table 7).

**Table 5:** Top diseases and biological functions associated with differentially methylated genes between smokers vs. non-smokers

Diseases and disorders	*P* value	No. of molecules
Hereditary disorder	1.44E-02–8.85E-05	3
Organismal injury and abnormalities	4.84E-02–8.85E-05	8
Renal and urological disease	2.82E-02–8.85E-05	2
Cancer	4.84E-02–3.80E-04	8
Cardiovascular disease	3.45E-02–3.803E-04	1
Molecular and cellular functions	*P* value	No. of molecules
Cell morphology	3.48E-02–3.80E-04	2
Cell-to-cell signaling and interaction	4.07E-02–3.80E-04	2
Cellular assembly and organization	4.44E-02–3.80E-04	3
Cell cycle	3.42E-03–7.61E-04	2
Cell death and survival	4.45E-02–7.61E-04	2
Physiological system development and function	*P* value	No. of molecules
Cardiovascular system development and function	4.07E-02–3.80E-04	3
Embryonic development	3.26E-02–3.80E-04	4
Lymphoid tissue structure and development	6.07E-02–3.80E-04	1
Organismal development	2.67E-02–3.80E-04	3
Organ morphology	2.52E-02–3.80E-04	3

**Table 6:** Top diseases and biological functions associated with differentially methylated genes between COPD vs. non-smokers

Diseases and disorders	*P* value	No. of molecules
Organismal injury and abnormalities	7.91E-03–1.41E-04	40
Cancer	7.91E-03–1.53E-04	32
Connective tissue disorders	7.91E-03–3.69E-04	8
Dermatological diseases and conditions	7.91E-03–5.12E-04	13
Respiratory disease	7.91E-03–5.43E-04	9
Molecular and cellular functions	*P* value	No. of molecules
Cellular development	7.91E-03–3.40E-10	58
Cell death and survival	7.91E-03–1.55E-07	49
Cell morphology	7.91E-03–3.15E-07	43
Cellular assembly and organization	7.91E-03–3.15E-07	32
Cellular function and maintenance	7.91E-03–3.15E-07	44
Physiological system development and function	*P* value	No. of molecules
Nervous system development and function	7.91E-03–3.33E-09	49
Tissue morphology	7.91E-03–2.60E-07	49
Tissue development	7.91E-03–3.15E-07	56
Organ morphology	7.91E-03–4.93E-07	32
Organismal development	7.91E-03–4.93E-07	60

**Table 7:** Canonical pathways in ingenuity pathway analysis associated with differentially methylated genes between smokers vs. non-smokers and COPD vs. non-smokers

Smokers vs. non-smokers		
Canonical pathways	*P* value	Overlap
B cell development	1.25E-02	3.0% 1/33
Antigen presentation pathway	1.40E-02	2.7% 1/37
Autoimmune thyroid disease signaling	1.77E-02	2.1% 1/47
Graft-vs.-host disease signaling	1.81E-02	2.1% 1/48
Nur77 signaling in T lymphocytes	2.15E-02	1.8% 1/57
COPD vs. non-smokers		
Canonical pathways	*P* value	Overlap
G Beta gamma signaling	5.35E-06	8.1% 7/86
GNRH signaling	6.42E-06	6.5% 8/124
Molecular mechanisms of cancer	6.98E-06	3.6% 13/366
Role of NFAT in cardiac hypertrophy	1.82E-05	4.8% 9/187
nNOS signaling in neurons	2.74E-05	11.1% 5/45

### Pyrosequencing analysis

Among the significant differentially methylated genes/probes identified in smokers vs. non-smokers and COPD vs. non-smokers based on their gene ontology (GO) analysis that directly relates to the biological functions, we chose a subset of genes for validation using pyrosequencing. In our pyrosequencing validation approach, the most significant subset of genes with CpG sites identified from our genome-wide DNA methylation analysis was selected based on their location (CpG island). It is evident that CpG island in a gene is associated with high CG density region (promoter or regulatory element), and the methylation status of which indirectly co-relates to its gene expression. Validating the differential methylation status of the specific subset of genes by pyrosequencing will further enhances the utility of those suggestive genes to be developed as a novel epigenetic signatures. Thus, DNA methylation can be used as a tool for the development of epigenetic-based diagnostic and therapeutic strategies in smokers and patients with COPD.

**Fig. 7:**
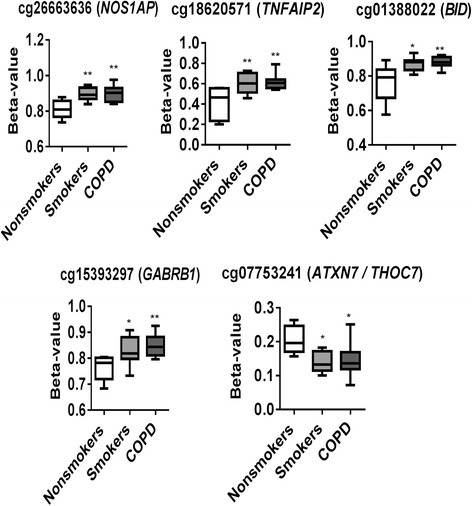
Differentially methylated CpG sites identified from genome-wide methylation profiling for pyrosequencing validation. Differential methylation analysis revealed CpG sites in genes significantly associated with smokers and COPD compared to non-smokers with a *P* value less than 0.05. Difference in mean beta values represents the difference in mean methylation between smokers and COPD compared to non-smokers (controls). The *y* axis represents the beta value. The gene symbol associated with CpG probes are provided in *parenthesis*. Data are represented as a *box plot* which displays the full range of variation (from min to max), the likely range of variation (the IQR) and a typical value (the median) for *n* = 8/group, and the significance determined using one-way ANOVA (Tukey’s multiple comparisons test). *P < 0.05, **P < 0.01, vs. non-smokers

**Fig. 8:**
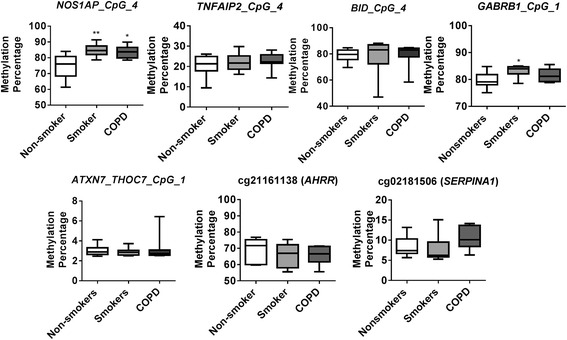
Pyrosequencing validation of CpG sites identified in genome-wide DNA methylation analysis. *NOS1AP* locus cg2663636, *TNFAIP2* locus cg18620571, *BID* locus cg01388022, *GABRB1* locus cg15393297, and *ATXN7* and *THOC7* locus cg07753241 were validated along with *AHRR* locus cg21161138 and *SERPINA1* locus cg02181506. *Boxplots* represent pyrosequencing methylation percentages between smokers and COPD compared to non-smokers control. Data are represented as *box plot* which displays the full range of variation (from min to max), the likely range of variation (the IQR) and a typical value (the median) for *n* = 8/group, and the significance determined using one-way ANOVA (Tukey’s multiple comparisons test). *P < 0.05, **P < 0.01, vs. non-smokers

In our genome-wide DNA methylation analysis, *NOS1AP* (cg26663636), *TNFAIP2* (cg18620571), *GABRB1* (cg15393297), and *BID* (cg01388022) are hyper-methylated both in smoker and COPD compared to non-smokers group (Fig. 7). We also selected *ATXN7* and *THOC7* genes (cg07753241) to validate since they both share the promoter (CpG island) that was hypo-methylated in smoker and COPD in our genome-wide DNA methylation analysis (Fig. 8). Besides *ATXN7* and *THOC7* all the other genes (*NOS1AP*, *TNFAIP2*, *GABRB1*, and *BID*) selected for pyrosequencing were significantly hyper-methylated in CpG island which is located in the gene body. Additionally, we included *AHRR* (cg21161138) [26] and *SERPINA1* (cg02181506) [11] which were shown to be significantly hypo-methylated in smokers and patients with COPD based on prior studies as part of the pyrosequencing validation in this study. We designed pyrosequencing primer sets for the above mentioned subset of genes (Additional file 1: Figure S3–S9 and Table 8). Of the five different genes validated by pyrosequencing, *NOS1AP* was significantly hyper-methylated in smokers and COPD compared to non-smokers group (Fig. 8). Interestingly, we found that most of the CpG sites (out of the 11 CpG sites) analyzed for *NOS1AP* showed significant hyper-methylation in smokers and COPD groups compared to non-smokers. Furthermore, when we combined the methylation percentage from all the 11 CpG sites for *NOS1AP*, we found significant hyper-methylation in smokers and COPD compared to the non-smokers group (Additional file 1: Figure S10). Pyrosequencing validations for *TNFAIP2* CpG site including additional 10 CpG sites assayed were not significant both in smokers and COPD (Additional file 1: Figure S11). Validation for *BID* CpG site was not significant in smokers and COPD compared to non-smokers groups. Instead, all the additional three CpG sites that were located close to the identified CpG site for *BID* showed significant hyper-methylation in smokers and COPD compared to the non-smokers group (Additional file 1: Figure S12). Our pyrosequencing analysis showed that the CpG site for *GABRB1* was significantly hyper-methylated in smokers, although not significant in COPD compared to the non-smokers group. All the additional CpG sites validated for *GABRB1* were not significant in smokers and COPD (Additional file 1: Figure S12). The CpG sites validated by pyrosequencing for *ATXN7* and *THOC7* for hypo-methylation in smokers and COPD compared to non-smokers group were not significant (Additional file 1: Figure S13).

**Table 8:** CpG pyrosequencing primer sequences

Gene	Forward primer 5′ to 3′	Reverse primer 3′ to 5′
*NOS1AP* (cg26663636)	TGTTTGGTGAAGTTGGAGTGT	^a^CCCAATTCCTACCTCTACAAAACATTCA
	Sequencing primer: GGTTTGGAGTTTATTAAGTT	
*TNFAIP2* (cg18620571)	GTTAGAGTAAGGTGGAGG	^a^CCAAATCCTCCTTCATAATAC
	Sequencing primer: AGAGTAAGGTGGAGG	
*BID* (cg01388022)	GAGTTAAATAATGGGTGTTTATGGAT	^a^TAACCCAACACTAACTATATCACCA
	Sequencing primer: ATGGAGAGAGTAGGT	
*GABRB1* (cg15393297)	^a^GTTAATGAAAATAGGTAAAGGTTTTGTAAT	AACCACTTATCTATAAAATTCACATC
	Sequencing primer: CTATCATAAAAATACATAATAACC	
*ATXN7/THOC7* (cg07753241)	^a^TAGGGGTTTTAGGGGAGGTTTAG	CTCCTCCTCCATAACTATTTACC
	Sequencing primer: ACTACATTATAAAAATTTAACCT	
*AHRR* (cg21161138)	GGTTGGTGGTGTAGGATATATT	^a^GACGGGACACCGCTGATCGTTTAACCCATCCTACCCAAATCCTAATAAT
	Sequencing primer: GGTTTTAGGTTTAGGGA	
*SERPINA1* (cg02181506)	TTTTGGTTTAGTTTAGGATTTTGAGG	^a^AAACACTATACCCAAAACATACACTAC
	Sequencing primer: GGATTTTGAGGGTTGTT	

^a^Biotin-labeled: biotinylated primer

## Discussion

Epigenetic modifications may play a critical role in regulating expression of genes involved in molecular pathways and cellular processes relevant to the pathogenesis of COPD [1]. Recently, we have reported cigarette smoke differentially regulates gene expression profiles of epigenetic chromatin modification enzymes and DNA methyltransferases in vitro in H292 cell and in vivo in mouse lung [27]. Previous genome-wide DNA methylation studies were focused on DNA methylation associated with smoking and COPD using various sample types, such as whole blood, lymphoblasts, pulmonary macrophages, sputum, buccal, small airway cells, and lung tissues [5–14, 17, 18]. In the present study, we used lung parenchymal tissue DNA to perform genome-wide DNA methylation analysis using the Illumina’s Infinium 450K methylation BeadChip and validated selective CpG sites from a subset of genes including *NOS1AP*, *BID*, and *GABRB1* by pyrosequencing analysis. We have identified several novel target CpG sites of specific genes by genome-wide methylation analysis using lung tissue DNA from smokers and patients with COPD based on their biological functions.

Pathway analysis revealed possible genes differentially methylated in our genome-wide study among the smokers and COPD group that belong to cardiovascular disease and respiratory disease, respectively, under the disease and disorders categories. Based on their molecular and cellular functions, the identified top differentially methylated probes (DMPs) among smokers include cell morphology, cell-to-cell signaling and interaction, cellular assembly and organization, cell cycle, and cell death and survival. Additionally, DMPs among the patients with COPD showed cellular development, cell death and survival, cell morphology, cellular assembly and organization, and cellular function and maintenance as their top molecular and cellular functions. Further, our pyrosequencing data from *NOS1AP*, *BID*, and *GABRB1* CpG sites analysis validates hyper-methylation in smokers and COPD compared to non-smokers. These genes are part of the relevant pathways in the pathogenesis of COPD, e.g., oxidative stress and mitochondrial dysfunction/autophagy. We speculate that altered DNA methylation status may affect gene expression of *NOS1AP*, *BID*, and *GABRB1* genes in lung tissues of smokers and patients with COPD and perturb the key cellular pathways involved in cellular senescence/autophagy/apoptosis. Future studies will address this issue by validating this hypothesis using the same samples for both DNA methylation and gene expression analysis.

It is known that promoter hyper-methylation can cause gene silencing. Hence, the results of our genome wide analysis may provide insights into the involvement of possible target genes in the pathogenesis of smoking-related chronic lung diseases by looking into which genes show changes in promoter DNA methylation. Among the top significant genes which are hyper-methylated in smokers include *ANKRD9*, *HLA-DRB5 TNFAIP2*, *ANGPT1*, *FAM8A1*, *ATXN7*, *THOC7*, *CNOT1*, and *ACTN4*. Similarly, when we compared COPD group with the non-smokers, all the top differentially methylated CpG sites significantly hyper-methylated includes *ANGPT1*, *PLD1*, *NFASC*, *ACTN4*, *RGS12*, *CAV1*, *PRKAG2*, *JARID2*, *HIPK2*, and *MECOM*. Some of the top differentially methylated genes, such as *ANKRD9*, *CAV1*, and *JARID2*, were previously known to be highly expressed in basal cells [28] significantly hyper-methylated in smokers and COPD groups confirmed by genome-wide DNA methylation analysis. A recent study suggests that caveolin-1 (Cav1) as a critical regulatory protein in pathological mechanisms of chronic inflammatory lung diseases. Loss of *CAV1* contributes to an imbalance in the Th17/Treg cell in patients with COPD [29]. Additionally, two other genes *TNFAIP2* and *ANGPT1* are identified to be hyper-methylated as smoking-responsive genes discovered by microarray and RNA sequencing approaches [30, 31]. Recently, CpG island hyper-methylation of *ANKRD18B* was associated with decreased expression of *ANKRD18B* in lung cancer tissues and cell lines compared to normal lung tissues [32]. Chi et al. reported that *ANKRD11* was one among the other genes (*ANKHD1* and *LGALS2*) with both regulatory DNA methylation sites in circulating monocytes and their mRNA expression that was associated with air pollution [33]. Another report showed *HLA-DRB5* gene (major histocompatibility complex, class II, DR beta 5) among the expression quantitative trait loci (eQTLs) expressed in neutrophils that may be involved in immune-recognition/regulation and mitochondrial function [34]. Wan et al. has shown association of genes such as *HIPK3*(homeodomain-interacting protein kinase 3), a serine-threonine protein kinase that participates in cAMP-mediated steroidogenesis involved in the synthesis and regulation of steroid hormones, was differentially methylated in COPD who were under systemic steroid use [9]. Furthermore, these CpG sites and loci differentially methylated in smokers and COPD can be used as epigenetic signature in smokers and COPD based on their methylation status. Overall, several gene families identified (i.e., *ANGPT*, *ANKRD*, *HIPK*, and *HLA-DRB*) among the top differentially methylated CpG sites from this study have been previously implicated as responsive genes in smokers and patients with COPD. This is based on their transcriptomics/DNA methylation data suggesting their role in the pathogenesis of smoking-related chronic lung diseases including COPD.

Our pyrosequencing data validates DNA methylation status of three candidate genes (*NOS1AP*, *BID*, and *GABRB1*) out of five genes that was identified from the genome-wide DNA methylation analysis. Nitric oxide synthase 1 adaptor protein (*NOS1AP*) is a cytosolic protein that binds to the signaling molecule nNOS (neuronal nitric oxide synthase) that plays an important role in relieving hemodynamic force, and thereby aids in protecting the arterial walls from vascular inflammation [35]. It is known that cardiovascular disease and co-morbidities are associated with COPD [36], COPD patients have a threefold risk of developing ischemic heart disease and lung cancer compared to smokers without clinical manifestation of COPD. These findings support that vascular dysfunction and systemic inflammation are linked to COPD [37, 38]. Interestingly, *NOS1AP* DNA methylation data from this study is in line with the previous report. Hyper-methylation of *NOS1AP* promoter is shown to be associated with intracranial aneurysm and brain arteriovenous malformation as a result of regular tobacco use [39] *NOS1AP* has been implicated in several human neurodegenerative diseases, such as cardiovascular disorders (stroke), psychiatric disorders, and post-traumatic stress disorders [40]. Variation among oxidant stress pathway genes including *NOS1AP* in recipients and donors has been shown to be associated with primary graft dysfunction after lung transplantation [41]. Earlier studies show association of *NOS1AP* differential methylation in smokers without any validation [42–44]. To our knowledge, ours is the first study to identify *NOS1AP* hyper-methylation in lung parenchymal tissues by genome-wide DNA methylation and pyrosequencing analysis showing association of nNOS with smokers and patients with COPD who are at risk to cardiovascular diseases (co-morbidities).

Recently, we and others have shown role of mitochondrial dysfunction (defective mitophagy) in cigarette smoke-induced cellular senescence in lung cells (epithelial and fibroblasts) in vitro and mouse lungs in vivo as well as in smokers and patients with COPD [45, 46]. *BID* (BH3-interacting domain death agonist) protein is a member of the *BCL-2* family of cell death regulators. *BID* functions in mitochondria for apoptosis during mitotic arrest [47] and engages a ROS-dependent, local inter-mitochondrial potentiation mechanism that amplifies the apoptotic signal [48]. *BID* CpG site was significantly hyper-methylated among the smokers and COPD group in the CpG island based on our genome-wide DNA methylation analysis. Our DNA methylation for *BID* CpG site directly or indirectly affirms the involvement of apoptotic mechanism including mitochondrial dysfunction role in the pathogenesis of COPD [49, 50].

*GABRB1* (gamma-aminobutyric acid type A receptor beta1 subunit) gene encodes for beta 1 subunit of the GABA A receptor, which is responsible for mediating inhibitory neurotransmission in the thalamus [51]. A previous report shows its role in alveolar fluid homeostasis of alveolar epithelial type II cells [52]. Gene expression changes caused by GABAergic system during smoking resulted in marked upregulation of *GAD67* expression in both the large and small airway epithelium of healthy smokers compared to healthy non-smokers. This altered *GAD67* expression was significantly correlated with increased *MUC5AC* gene expression during smoking [53]. Our genome-wide DNA methylation data show significant hyper-methylation of *GABRB1* in smokers and COPD. Nicotine can affect gene expression of DNA methyltransferase 1 (*DNMT1*) that directly or indirectly influences the promoter methylation status of GABAergic neurons, thereby providing a plausible link to nicotine addiction [54]. However, it remains unclear how *GABRB1* functions as ligand-gated chloride channel during cigarette smoke-induced pulmonary toxic responses in the lungs of smokers and patients with COPD. Likewise, understanding the DNA methylation signature in lung tissues of smokers and COPD patients will enable us to discover the possibility of using selective epigenetic-based drugs that can attenuate progression of COPD in smokers.

In this study, we also included Serpin family A member 1 (*SERPINA1*) CpG site (cg02181506) and aryl hydrocarbon receptor repressor (*AHRR*) CpG site (cg21161138) for analysis along with the other CpG sites for validation even though they were not identified in our genome-wide methylation analysis. Surprisingly, our pyrosequencing data for *SERPINA1* and *AHRR* hypo-methylation in smokers and patients with COPD did not show significant changes. *SERPINA1* encodes for alpha-1-antitrypsin deficiency that contributes to the genetic susceptibility of COPD. Previously, *SERPINA1* hypo-methylation using whole blood DNA has been shown in patients with COPD correlated with lower lung function [11]. In this study, pyrosequencing analysis for *SERPINA1* showed correlation in the methylation status (methylation percentages) among the non-smoker groups. However, we were unable to see any significant hypo-methylation of *SERPINA1* CpG site in smokers and COPD groups compared to non-smokers. Similarly, smoking-induced DNA methylation alteration in *AHRR* has been evidently documented using DNA from lymphoblasts, alveolar macrophages, WBCs, and whole blood, suggesting the role of epigenetic effects mediated by CS on carcinogenesis and other related co-morbidities in a tissue-independent manner [6, 26, 55–58]. These studies have implicated the role of CS-mediated oxidative stress and inflammatory response associated with DNA methylation (hypo-methylation) in *SERPINA1* and *AHRR* CpG sites. Our observations regarding the lack of correlation observed in *SERPINA1* and *AHRR* hypo-methylation in smokers and COPD could be due to limited sample size, tissue heterogeneity (cell-type present in lung parenchymal tissue), and genetic variants present in our samples. We were unable to validate DNA methylation changes of the *TNFAIP2* and *ATXN7/THOC* genes using pyrosequencing. The exact reason for lack on reproducibility of findings is not known. This inconsistency shows that validations are important to confirm the array-based DNA methylation analysis. Therefore, it is important to validate the identified CpG sites using another technique which is very specific for the target CpG sites, such as the pyrosequencing assays as used in this study.

In this study, we also identified DMR of oxidative stress-related genes, steroid-responsive genes, and cellular signaling genes linked to molecular and cellular functions (cell death and survival pathways). Previous studies have provided evidence that cigarette smoking plays an important role in epigenetic regulation of oxidative stress genes (e.g., glutamate-cysteine ligase catalytic subunit (GCLC)) and immune-related genes (*IL-12RB2* and *WIF-1*) involved in the pathogenesis of COPD and lung cancer (*CCDC37*, and *MAP1B*) [5, 11, 59–61]. Earlier studies have shown site-specific DNA methylation during steroid resistance in COPD, in smokers even after cessation (e.g., Factor II receptor-like 3 (*F2RL3*) and G-protein-coupled receptor 15 (*GPR15*)), and in newborns affected by maternal smoking (in utero, or early life exposure) leading to complications later in life [9, 10, 16]. Similarly, promoter methylation of *p16* (*CDKN2A*) and *GATA4* in sputum DNA from smokers showed significant correlation with decline in lung function [7]. Based on our observations and prior studies, we suggest DNA methylation does play an important role in regulation of oxidative stress, pro-inflammatory responses, and systemic steroid exposure in COPD [5, 9, 12].

Our study has some limitations, such as limited number of human lung tissues used and cellular heterogeneity/specific lung cell types which are affected by chronic cigarette smoking including the statistical analysis used for genome-wide DNA methylation analysis. However, this study is unique as it deals with parenchymal lung tissues which are involved in the pathogenesis of COPD. Further, our global DNA methylation data are indeed validated by pyrosequencing for the identified target genes. Future studies will additionally validate the identified genes using a larger cohort so as to determine the utility of suggestive genes as novel epigenetic signatures in smokers and patients with COPD.

## Conclusions

In conclusion, we found novel genes/pathways associated with possible differential DNA methylation in smokers and patients with COPD using the Infinium HumanMethylation450 BeadChip. Based on genome-wide DNA methylation and functional network analysis, selective CpG sites (*NOS1AP*, *BID*, and *GABRB1*) differentially methylated in smokers and COPD compared to non-smokers were validated by pyrosequencing. However, future studies will further validate differential methylation of target genes and their gene expression signatures in lung tissues from non-smokers, smokers, and patients with COPD. Overall, some of the top differentially methylated probes/genes identified in this study may be used as lung tissue-specific epigenetic signatures to stratify smoking exposure and predict the risk of developing smoking-related chronic lung diseases including COPD, COPD-exacerbations, and lung cancer after further validation of the suggested genes in a larger cohort. Further, a comprehensive, systematic assembly of COPD-related gene deregulation is required to integrate existing epigenome-wide DNA methylome and transcriptome signatures as they relate to COPD phenotypes and ultimately merge these data across platforms into a systems biology paradigm in COPD pathogenesis and its exacerbations.

## Additional files

Additional file 1: Figures S1-S13. **Figure S1.**Boxplot shows distribution of pre-processed DNA methylation level (β values) from all the samples and different groups used for comparison. **Figure S2.**Venn diagrams shows DNA methylation loci that were identified to be overlapping and or shared between Non-smokers vs. Smokers compared to Non-smokers vs. COPD and Smoker vs. COPD. **Figure S3-S9.** Target sequence for the *NOS1AP* (cg26663636), *TNFAIP2* (cg18620571), *BID *(cg01388022), *GABRB1* (cg15393297), *ATXN7/THOC7* (cg07753241), *AHRR *(cg21161138) and *SERPINA1* (cg02181506) amplicons. **Figure S10-13.** Pyrosequencing validation of additional CpG sites for *NOS1AP*, *TNFAIP2*, *BID*, and *ATXN7/THOC7*. (PPTX 1.93 mb)

Additional file 2: Table S1. CpGs differentially methylated in lung DNA in relation to smokers vs. non-smokers (*P* < 0.001). (CSV 1 kb)

Additional file 3: Table S2. CpGs differentially methylated in lung DNA in relation to COPD vs. non-smokers (*P* < 0.001). (CSV 37 kb)

Additional file 4: Table S3. CpGs differentially methylated in lung DNA in relation to COPD vs. smokers (*P* < 0.001). (CSV 1 kb)

Additional file 5: Table S4. CpGs differentially methylated in lung DNA in relation to smokers vs. non-smokers (*P* < 0.01). (CSV 16 kb)

Additional file 6: Table S5. CpGs differentially methylated in lung DNA in relation to COPD vs. non-smokers (*P* < 0.01). (CSV 273 kb)

Additional file 7: Table S6. CpGs differentially methylated in lung DNA in relation to COPD vs. smokers (*P* < 0.01). (CSV 19 kb)

Additional file 8: Table S7. CpGs differentially methylated in lung DNA in relation to smokers vs. non-smokers (*P* < 0.05). (CSV 1487 kb)

Additional file 9: Table S8. CpGs differentially methylated in lung DNA in relation to COPD vs. non-smokers (*P* < 0.05). (CSV 4866 kb)

Additional file 10: Table S9. CpGs differentially methylated in lung DNA in relation to COPD vs. smokers (*P* < 0.05). (CSV 1597 kb)

Additional file 11: Table S10. Top 100 CpGs differentially methylated along with five CpG sites chosen for validation in lung DNA in relation to smokers vs. non-smokers shown in heatmap (Fig. 3a). (CSV 2 kb)

Additional file 12: Table S11. Top 100 CpGs differentially methylated along with five CpG sites chosen for validation in lung DNA in relation to COPD vs. non-smokers shown in heatmap (Fig. 3b). (CSV 2 kb)

Additional file 13: Table S12. Top 100 CpGs differentially methylated along with five CpG sites chosen for validation in lung DNA in relation to COPD vs. smokers shown in heatmap (Fig. 3c). (CSV 3 kb)

## Abbreviations

ACTN4: Actinin alpha 4
AHRR: Aryl hydrocarbon receptor repressor
AML: Acute myeloid leukemia
ANGPT1: Angiopoietin 1
ANKHD1: Ankyrin repeat and KH domain containing 1
ANKRD9: Ankyrin repeat domain 9
ATXN7: Ataxin 7
BID: BH3 interacting domain death agonist
CAV1: Caveolin 1
CCDC37: Coiled-coil domain containing 37
CNOT1: CCR4-NOT transcription complex subunit 1
COPD: Chronic obstructive pulmonary disease
CpG: Cytosine-phosphate-guanine
CS: Cigarette smoke
CSMD1: CUB and sushi multiple domains 1
DMBA: 7, 12-Dimethylbenz[a]anthracene
DMP: Differentially methylated probe
DNMT1: DNA methyltransferase 1
F2RL3: F2R-like thrombin/trypsin receptor 3
FAM8A1: Family with sequence similarity 8 member A1
FEV1: Forced expiratory volume in 1 s
GABRB1: Gamma-aminobutyric acid type A receptor beta1 subunit
GAD67: Glutamate decarboxylase 67
GCLC: Glutamate-cysteine ligase catalytic subunit
GO: Gene ontology
GPR15: G protein-coupled receptor 15
HIPK2: Homeodomain interacting protein kinase 2
HIPK3: Homeodomain interacting protein kinase 3
HLA-DRB5: Major histocompatibility complex, class II, DR beta 5
IL12RB2: Interleukin 12 receptor subunit beta 2
IMA: Illumina methylation analyzer
IPA: Ingenuity pathway analysis
JARID2: Jumonji and AT-rich interaction domain containing 2
LGALS2: Galectin 2
MAP1B: Microtubule associated protein 1B
MECOM: MDS1 and EVI1 complex locus
MUC5AC: Mucin 5AC, oligomeric mucus/gel-forming
NFASC: Neurofascin
NOS1AP: Nitric oxide synthase 1 adaptor protein
PLD1: Phospholipase D1
PRKAG2: Protein kinase AMP-activated non-catalytic subunit gamma 2
PRKCA: Protein kinase C alpha
PRKCABP: Protein kinase C, alpha binding protein
RGS12: Regulator of G-protein signaling 12
ROS: Reactive oxygen species
SERPINA1: Serpin family A member 1
SNP: Single-nucleotide polymorphism
THOC7: THO complex 7
TNFAIP2: TNF alpha induced protein 2
WBC: White blood cells
WIF1: WNT inhibitory factor 1
