# Supplementary material for: DNA methylation profiling in peripheral lung tissues of smokers and patients with COPD
Source: Clin Epigenetics. 2017 Apr 14;9:38. doi: 10.1186/s13148-017-0335-5 (PMC5391602; doi:10.1186/s13148-017-0335-5)
Supplement: Supplementary file 1 — Figure S1.Boxplot shows distribution of pre-processed DNA methylation level (β values) from all the samples and different groups used for comparison. Figure S2.Venn diagrams shows DNA methylation loci that were identified to be overlapping and or shared between Non-smokers vs. Smokers compared to Non-smokers vs. COPD and Smoker vs. COPD. Figure S3-S9. Target sequence for the NOS1AP (cg26663636), TNFAIP2 (cg18620571), BID (cg01388022), GABRB1 (cg15393297), ATXN7/THOC7 (cg07753241), AHRR (cg21161138) and SERPINA1 (cg02181506) amplicons. Figure S10-13. Pyrosequencing validation of additional CpG sites for NOS1AP, TNFAIP2, BID, and ATXN7/THOC7. (PPTX 1.93 mb) [file 13148_2017_335_MOESM1_ESM.pptx]

## Slide 1
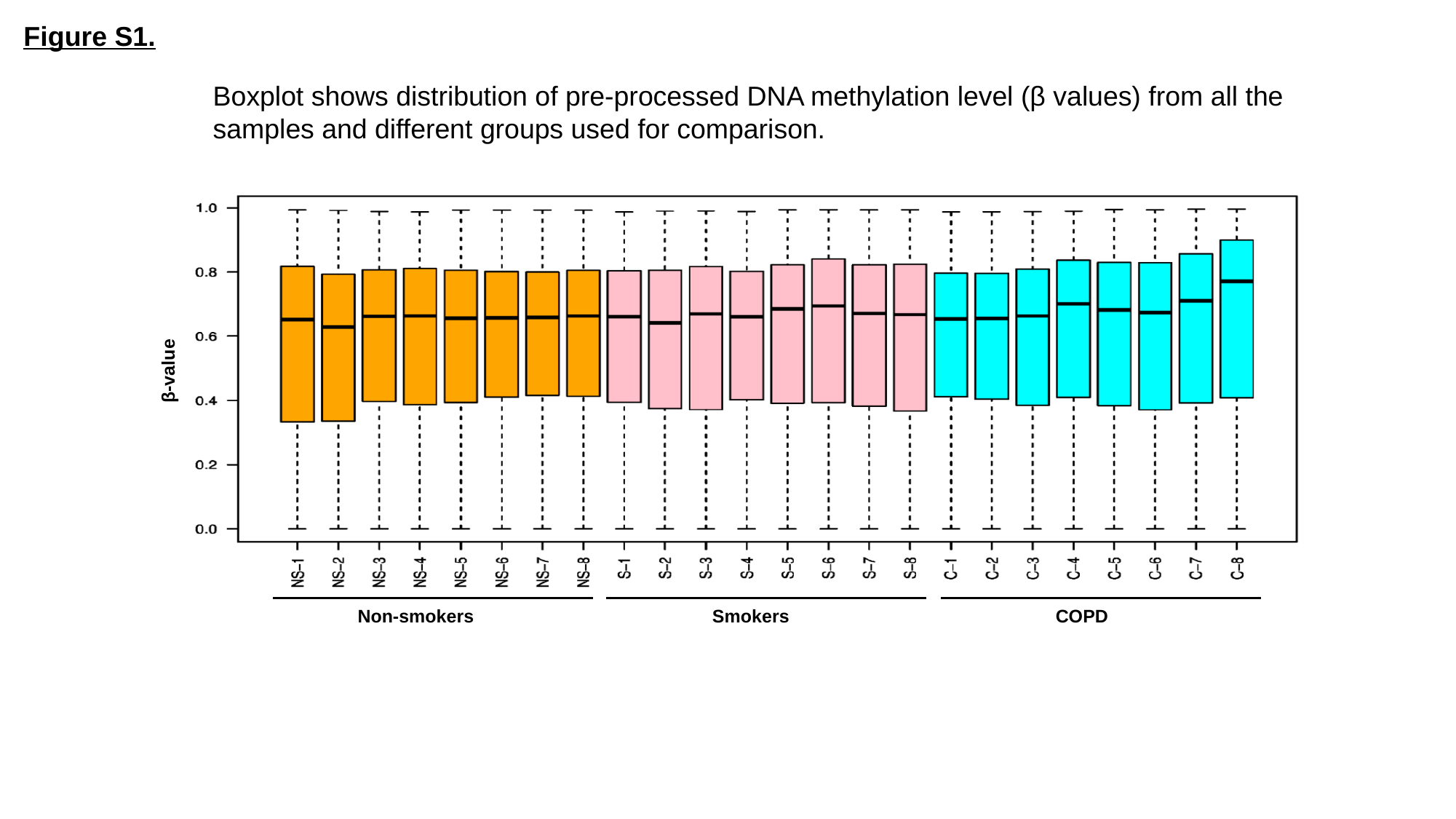

Figure S1.
Boxplot shows distribution of pre-processed DNA methylation level (β values) from all the samples and different groups used for comparison.
β-value
Smokers
COPD
Non-smokers

## Slide 2
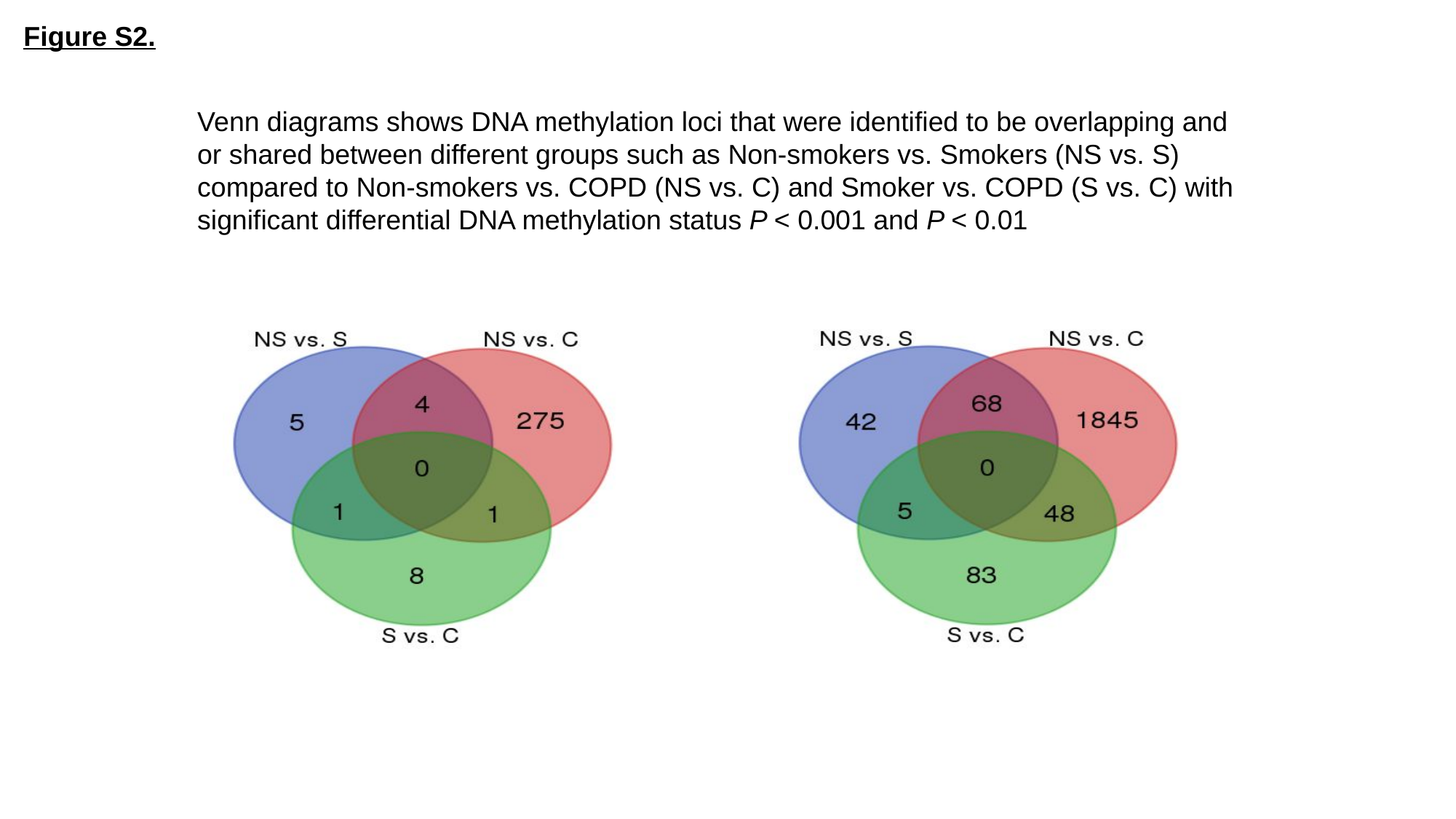

Figure S2.
Venn diagrams shows DNA methylation loci that were identified to be overlapping and or shared between different groups such as Non-smokers vs. Smokers (NS vs. S) compared to Non-smokers vs. COPD (NS vs. C) and Smoker vs. COPD (S vs. C) with significant differential DNA methylation status P < 0.001 and P < 0.01

## Slide 3
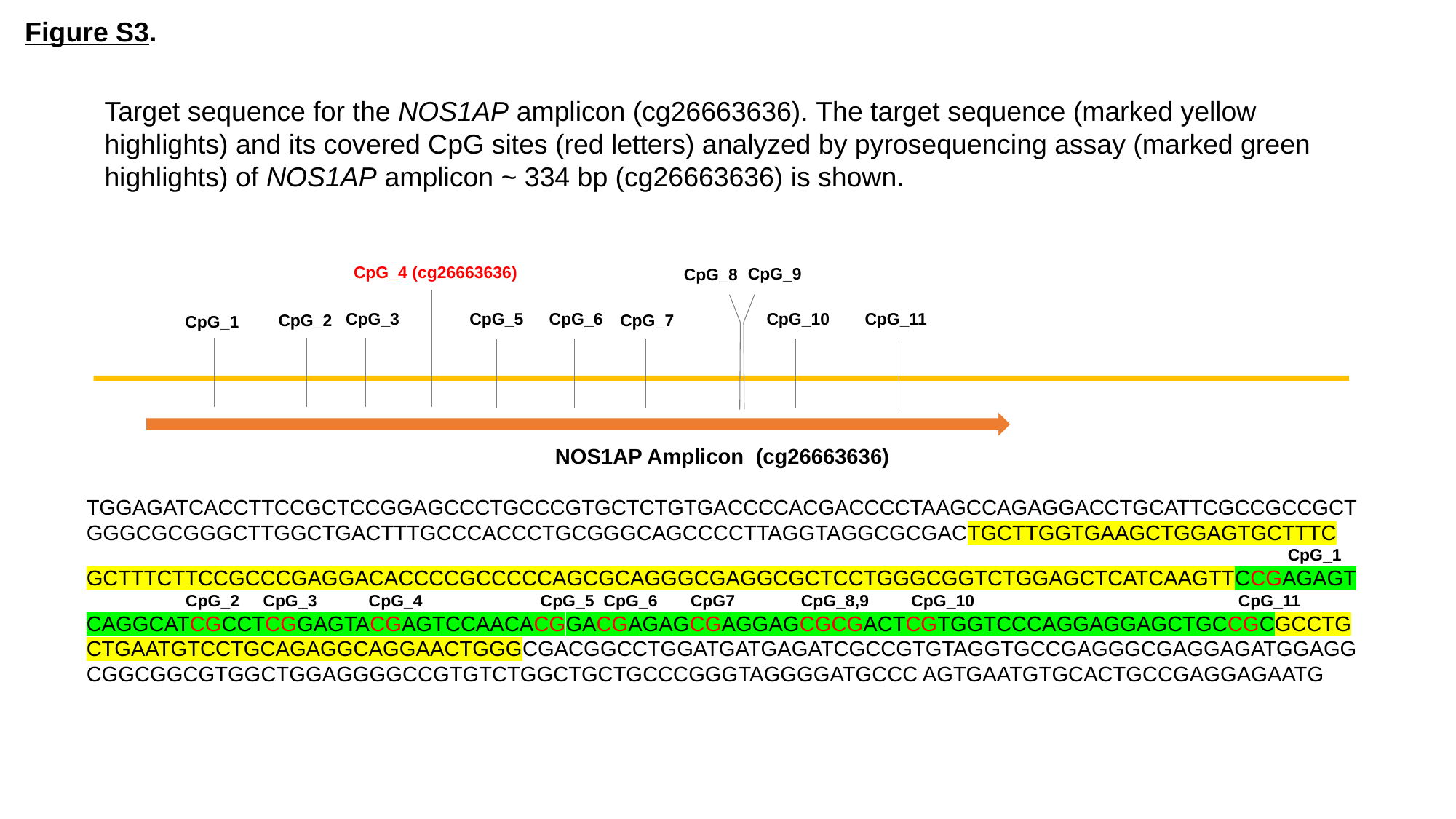

Figure S3.
Target sequence for the NOS1AP amplicon (cg26663636). The target sequence (marked yellow highlights) and its covered CpG sites (red letters) analyzed by pyrosequencing assay (marked green highlights) of NOS1AP amplicon ~ 334 bp (cg26663636) is shown.
CpG_4 (cg26663636)
CpG_9
CpG_8
CpG_10
CpG_11
CpG_3
CpG_5
CpG_6
CpG_2
CpG_7
CpG_1
NOS1AP Amplicon (cg26663636)
TGGAGATCACCTTCCGCTCCGGAGCCCTGCCCGTGCTCTGTGACCCCACGACCCCTAAGCCAGAGGACCTGCATTCGCCGCCGCTGGGCGCGGGCTTGGCTGACTTTGCCCACCCTGCGGGCAGCCCCTTAGGTAGGCGCGACTGCTTGGTGAAGCTGGAGTGCTTTC
 CpG_1
GCTTTCTTCCGCCCGAGGACACCCCGCCCCCAGCGCAGGGCGAGGCGCTCCTGGGCGGTCTGGAGCTCATCAAGTTCCGAGAGT
 CpG_2 CpG_3 CpG_4 CpG_5 CpG_6 CpG7 CpG_8,9 CpG_10 CpG_11
CAGGCATCGCCTCGGAGTACGAGTCCAACACGGACGAGAGCGAGGAGCGCGACTCGTGGTCCCAGGAGGAGCTGCCGCGCCTGCTGAATGTCCTGCAGAGGCAGGAACTGGGCGACGGCCTGGATGATGAGATCGCCGTGTAGGTGCCGAGGGCGAGGAGATGGAGGCGGCGGCGTGGCTGGAGGGGCCGTGTCTGGCTGCTGCCCGGGTAGGGGATGCCC AGTGAATGTGCACTGCCGAGGAGAATG

## Slide 4
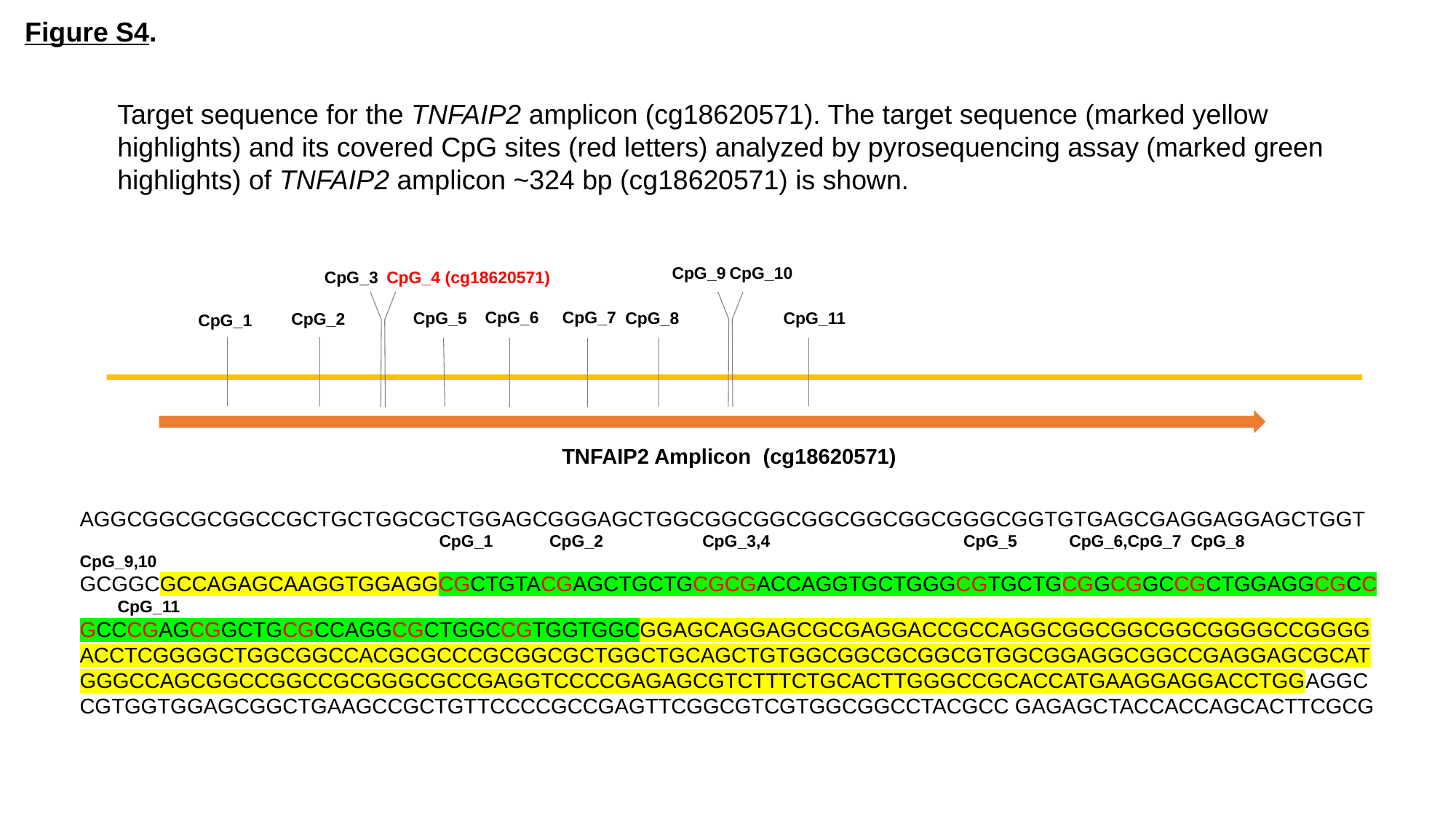

Figure S4.
Target sequence for the TNFAIP2 amplicon (cg18620571). The target sequence (marked yellow highlights) and its covered CpG sites (red letters) analyzed by pyrosequencing assay (marked green highlights) of TNFAIP2 amplicon ~324 bp (cg18620571) is shown.
CpG_9
CpG_10
CpG_3
CpG_4 (cg18620571)
CpG_6
CpG_7
CpG_5
CpG_8
CpG_11
CpG_2
CpG_1
TNFAIP2 Amplicon (cg18620571)
AGGCGGCGCGGCCGCTGCTGGCGCTGGAGCGGGAGCTGGCGGCGGCGGCGGCGGCGGGCGGTGTGAGCGAGGAGGAGCTGGT
 CpG_1 CpG_2 CpG_3,4 CpG_5 CpG_6,CpG_7 CpG_8 CpG_9,10
GCGGCGCCAGAGCAAGGTGGAGGCGCTGTACGAGCTGCTGCGCGACCAGGTGCTGGGCGTGCTGCGGCGGCCGCTGGAGGCGCC
 CpG_11
GCCCGAGCGGCTGCGCCAGGCGCTGGCCGTGGTGGCGGAGCAGGAGCGCGAGGACCGCCAGGCGGCGGCGGCGGGGCCGGGGACCTCGGGGCTGGCGGCCACGCGCCCGCGGCGCTGGCTGCAGCTGTGGCGGCGCGGCGTGGCGGAGGCGGCCGAGGAGCGCATGGGCCAGCGGCCGGCCGCGGGCGCCGAGGTCCCCGAGAGCGTCTTTCTGCACTTGGGCCGCACCATGAAGGAGGACCTGGAGGCCGTGGTGGAGCGGCTGAAGCCGCTGTTCCCCGCCGAGTTCGGCGTCGTGGCGGCCTACGCC GAGAGCTACCACCAGCACTTCGCG

## Slide 5
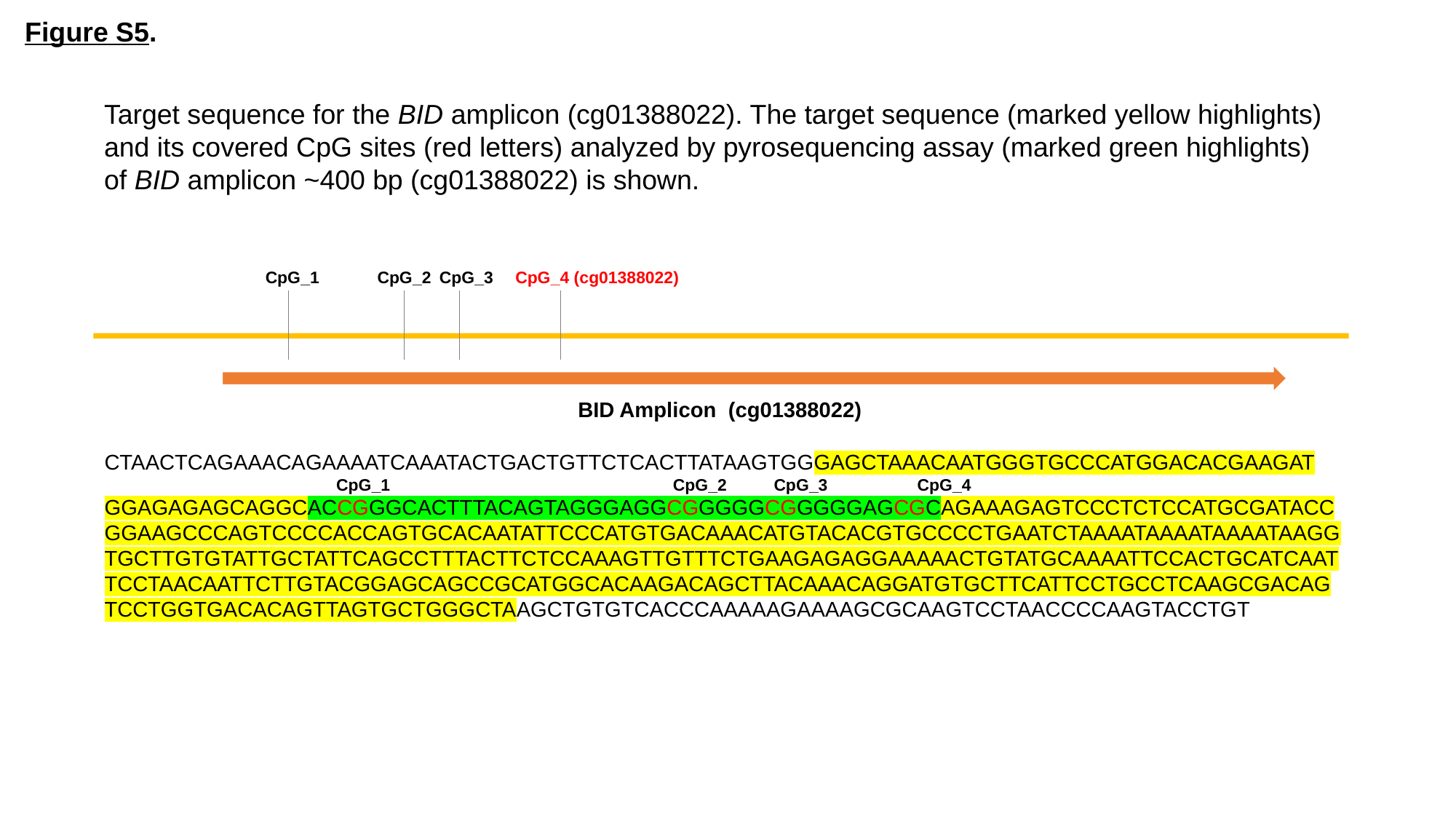

Figure S5.
Target sequence for the BID amplicon (cg01388022). The target sequence (marked yellow highlights) and its covered CpG sites (red letters) analyzed by pyrosequencing assay (marked green highlights) of BID amplicon ~400 bp (cg01388022) is shown.
CpG_2
CpG_3
CpG_4 (cg01388022)
CpG_1
BID Amplicon (cg01388022)
CTAACTCAGAAACAGAAAATCAAATACTGACTGTTCTCACTTATAAGTGGGAGCTAAACAATGGGTGCCCATGGACACGAAGAT
 CpG_1 CpG_2 CpG_3 CpG_4
GGAGAGAGCAGGCACCGGGCACTTTACAGTAGGGAGGCGGGGGCGGGGGAGCGCAGAAAGAGTCCCTCTCCATGCGATACCGGAAGCCCAGTCCCCACCAGTGCACAATATTCCCATGTGACAAACATGTACACGTGCCCCTGAATCTAAAATAAAATAAAATAAGGTGCTTGTGTATTGCTATTCAGCCTTTACTTCTCCAAAGTTGTTTCTGAAGAGAGGAAAAACTGTATGCAAAATTCCACTGCATCAATTCCTAACAATTCTTGTACGGAGCAGCCGCATGGCACAAGACAGCTTACAAACAGGATGTGCTTCATTCCTGCCTCAAGCGACAGTCCTGGTGACACAGTTAGTGCTGGGCTAAGCTGTGTCACCCAAAAAGAAAAGCGCAAGTCCTAACCCCAAGTACCTGT

## Slide 6
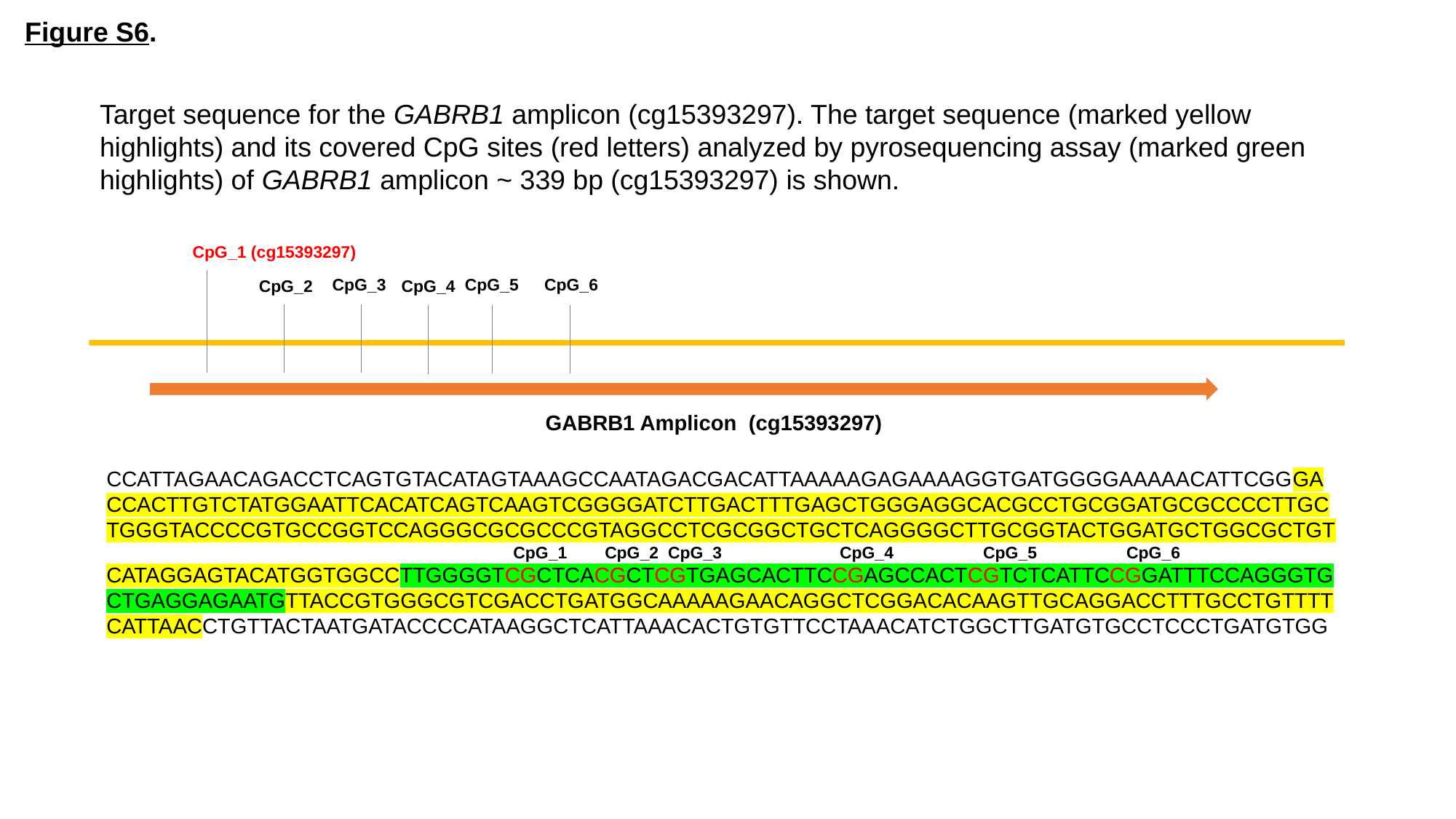

Figure S6.
Target sequence for the GABRB1 amplicon (cg15393297). The target sequence (marked yellow highlights) and its covered CpG sites (red letters) analyzed by pyrosequencing assay (marked green highlights) of GABRB1 amplicon ~ 339 bp (cg15393297) is shown.
CpG_1 (cg15393297)
CpG_3
CpG_5
CpG_6
CpG_2
CpG_4
GABRB1 Amplicon (cg15393297)
CCATTAGAACAGACCTCAGTGTACATAGTAAAGCCAATAGACGACATTAAAAAGAGAAAAGGTGATGGGGAAAAACATTCGGGACCACTTGTCTATGGAATTCACATCAGTCAAGTCGGGGATCTTGACTTTGAGCTGGGAGGCACGCCTGCGGATGCGCCCCTTGCTGGGTACCCCGTGCCGGTCCAGGGCGCGCCCGTAGGCCTCGCGGCTGCTCAGGGGCTTGCGGTACTGGATGCTGGCGCTGT
 CpG_1 CpG_2 CpG_3 CpG_4 CpG_5 CpG_6
CATAGGAGTACATGGTGGCCTTGGGGTCGCTCACGCTCGTGAGCACTTCCGAGCCACTCGTCTCATTCCGGATTTCCAGGGTGCTGAGGAGAATGTTACCGTGGGCGTCGACCTGATGGCAAAAAGAACAGGCTCGGACACAAGTTGCAGGACCTTTGCCTGTTTTCATTAACCTGTTACTAATGATACCCCATAAGGCTCATTAAACACTGTGTTCCTAAACATCTGGCTTGATGTGCCTCCCTGATGTGG

## Slide 7
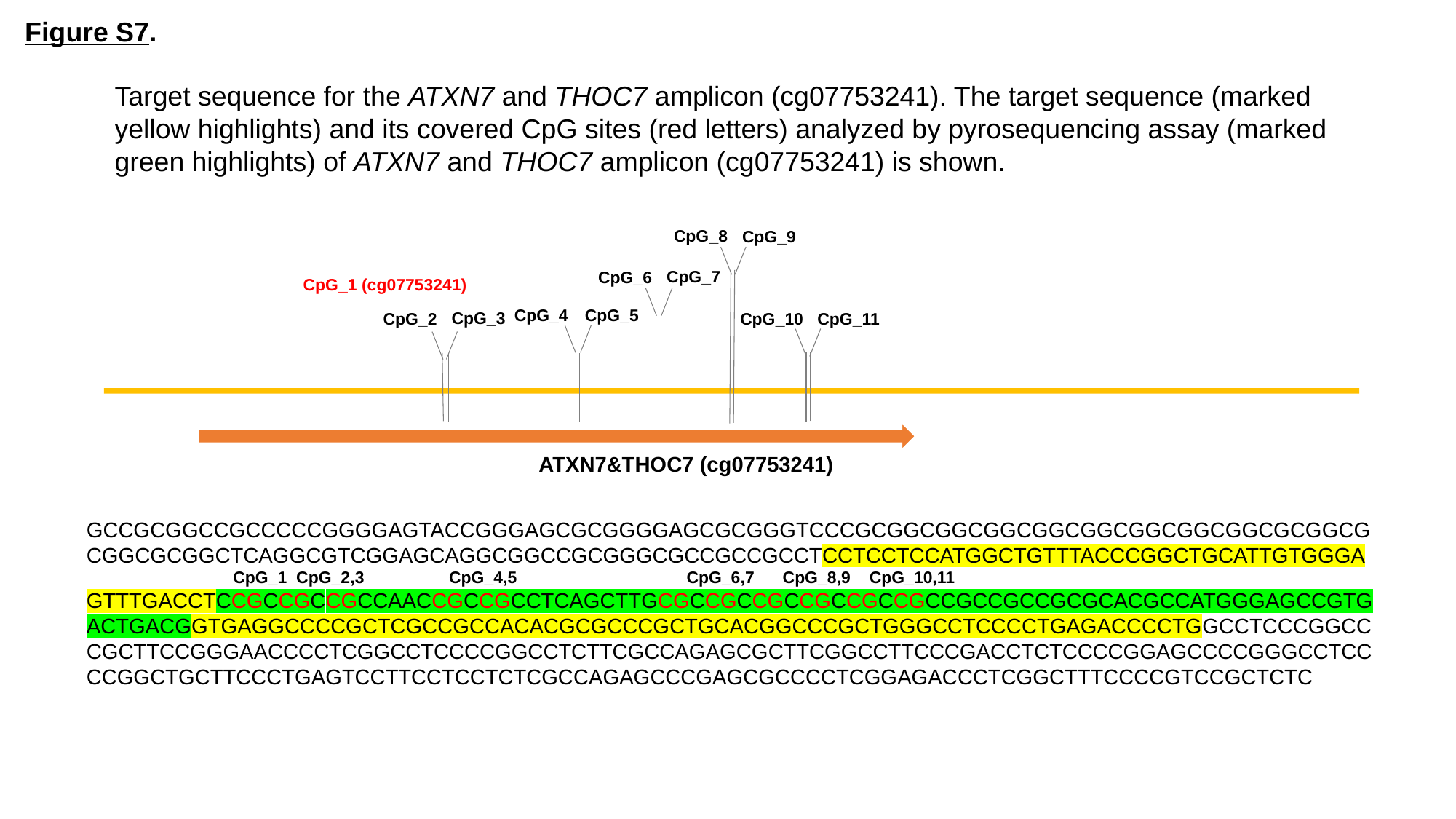

Figure S7.
Target sequence for the ATXN7 and THOC7 amplicon (cg07753241). The target sequence (marked yellow highlights) and its covered CpG sites (red letters) analyzed by pyrosequencing assay (marked green highlights) of ATXN7 and THOC7 amplicon (cg07753241) is shown.
CpG_8
CpG_9
CpG_7
CpG_6
CpG_1 (cg07753241)
CpG_5
CpG_4
CpG_3
CpG_10 CpG_11
CpG_2
ATXN7&THOC7‍ (cg07753241)
GCCGCGGCCGCCCCCGGGGAGTACCGGGAGCGCGGGGAGCGCGGGTCCCGCGGCGGCGGCGGCGGCGGCGGCGGCGCGGCGCGGCGCGGCTCAGGCGTCGGAGCAGGCGGCCGCGGGCGCCGCCGCCTCCTCCTCCATGGCTGTTTACCCGGCTGCATTGTGGGA
 CpG_1 CpG_2,3 CpG_4,5 CpG_6,7 CpG_8,9 CpG_10,11
GTTTGACCTCCGCCGCCGCCAACCGCCGCCTCAGCTTGCGCCGCCGCCGCCGCCGCCGCCGCCGCGCACGCCATGGGAGCCGTGACTGACGGTGAGGCCCCGCTCGCCGCCACACGCGCCCGCTGCACGGCCCGCTGGGCCTCCCCTGAGACCCCTGGCCTCCCGGCCCGCTTCCGGGAACCCCTCGGCCTCCCCGGCCTCTTCGCCAGAGCGCTTCGGCCTTCCCGACCTCTCCCCGGAGCCCCGGGCCTCCCCGGCTGCTTCCCTGAGTCCTTCCTCCTCTCGCCAGAGCCCGAGCGCCCCTCGGAGACCCTCGGCTTTCCCCGTCCGCTCTC

## Slide 8
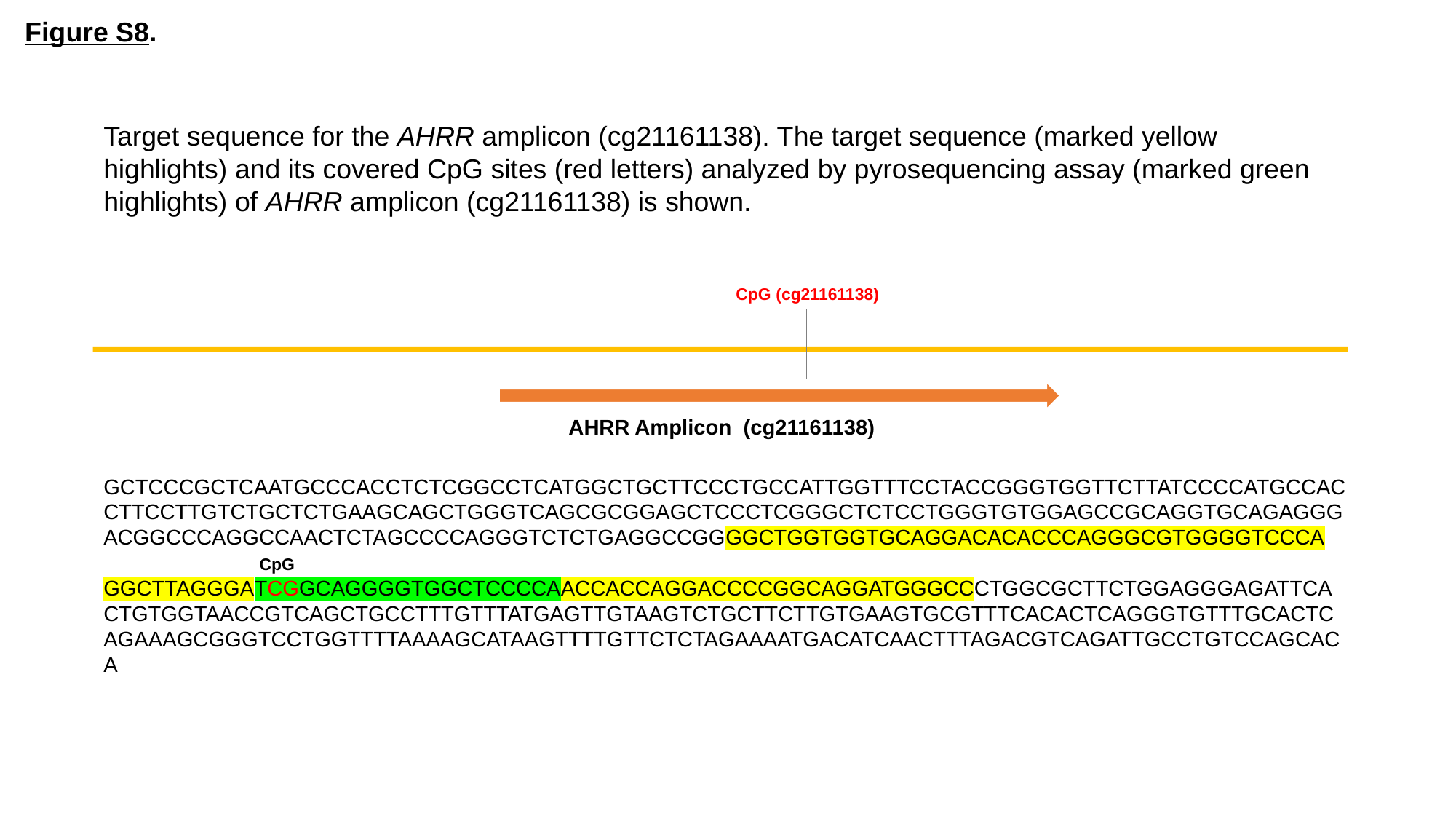

Figure S8.
Target sequence for the AHRR amplicon (cg21161138). The target sequence (marked yellow highlights) and its covered CpG sites (red letters) analyzed by pyrosequencing assay (marked green highlights) of AHRR amplicon (cg21161138) is shown.
CpG (cg21161138)
AHRR Amplicon (cg21161138)
GCTCCCGCTCAATGCCCACCTCTCGGCCTCATGGCTGCTTCCCTGCCATTGGTTTCCTACCGGGTGGTTCTTATCCCCATGCCACCTTCCTTGTCTGCTCTGAAGCAGCTGGGTCAGCGCGGAGCTCCCTCGGGCTCTCCTGGGTGTGGAGCCGCAGGTGCAGAGGGACGGCCCAGGCCAACTCTAGCCCCAGGGTCTCTGAGGCCGGGGCTGGTGGTGCAGGACACACCCAGGGCGTGGGGTCCCA
 CpG
GGCTTAGGGATCGGCAGGGGTGGCTCCCCAACCACCAGGACCCCGGCAGGATGGGCCCTGGCGCTTCTGGAGGGAGATTCACTGTGGTAACCGTCAGCTGCCTTTGTTTATGAGTTGTAAGTCTGCTTCTTGTGAAGTGCGTTTCACACTCAGGGTGTTTGCACTCAGAAAGCGGGTCCTGGTTTTAAAAGCATAAGTTTTGTTCTCTAGAAAATGACATCAACTTTAGACGTCAGATTGCCTGTCCAGCACA

## Slide 9
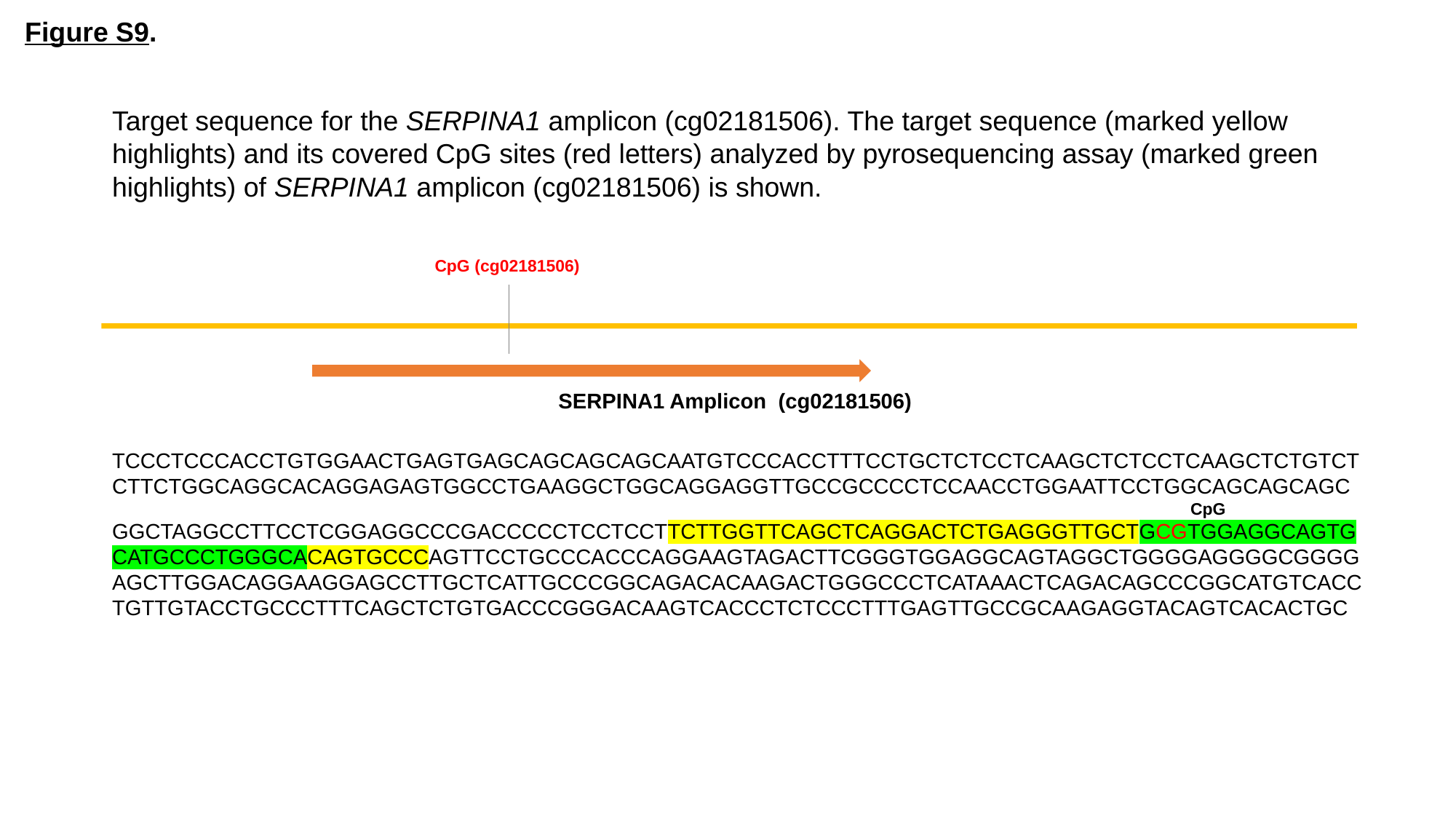

Figure S9.
Target sequence for the SERPINA1 amplicon (cg02181506). The target sequence (marked yellow highlights) and its covered CpG sites (red letters) analyzed by pyrosequencing assay (marked green highlights) of SERPINA1 amplicon (cg02181506) is shown.
CpG (cg02181506)
SERPINA1 Amplicon (cg02181506)
TCCCTCCCACCTGTGGAACTGAGTGAGCAGCAGCAGCAATGTCCCACCTTTCCTGCTCTCCTCAAGCTCTCCTCAAGCTCTGTCTCTTCTGGCAGGCACAGGAGAGTGGCCTGAAGGCTGGCAGGAGGTTGCCGCCCCTCCAACCTGGAATTCCTGGCAGCAGCAGC
 CpG
GGCTAGGCCTTCCTCGGAGGCCCGACCCCCTCCTCCTTCTTGGTTCAGCTCAGGACTCTGAGGGTTGCTGCGTGGAGGCAGTGCATGCCCTGGGCACAGTGCCCAGTTCCTGCCCACCCAGGAAGTAGACTTCGGGTGGAGGCAGTAGGCTGGGGAGGGGCGGGGAGCTTGGACAGGAAGGAGCCTTGCTCATTGCCCGGCAGACACAAGACTGGGCCCTCATAAACTCAGACAGCCCGGCATGTCACCTGTTGTACCTGCCCTTTCAGCTCTGTGACCCGGGACAAGTCACCCTCTCCCTTTGAGTTGCCGCAAGAGGTACAGTCACACTGC

## Slide 10
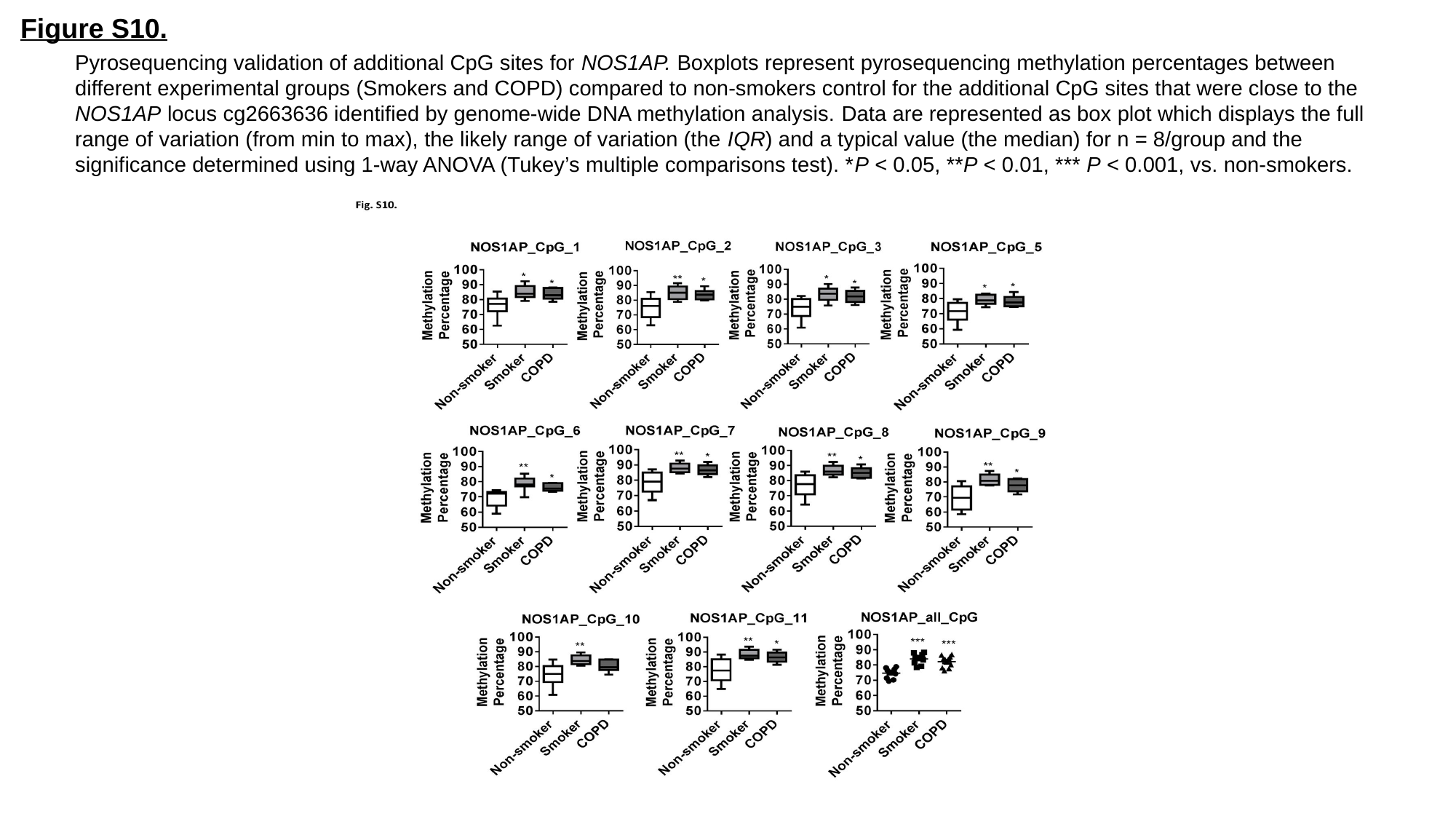

Figure S10.
Pyrosequencing validation of additional CpG sites for NOS1AP. Boxplots represent pyrosequencing methylation percentages between different experimental groups (Smokers and COPD) compared to non-smokers control for the additional CpG sites that were close to the NOS1AP locus cg2663636 identified by genome-wide DNA methylation analysis. Data are represented as box plot which displays the full range of variation (from min to max), the likely range of variation (the IQR) and a typical value (the median) for n = 8/group and the significance determined using 1-way ANOVA (Tukey’s multiple comparisons test). *P < 0.05, **P < 0.01, *** P < 0.001, vs. non-smokers.

## Slide 11
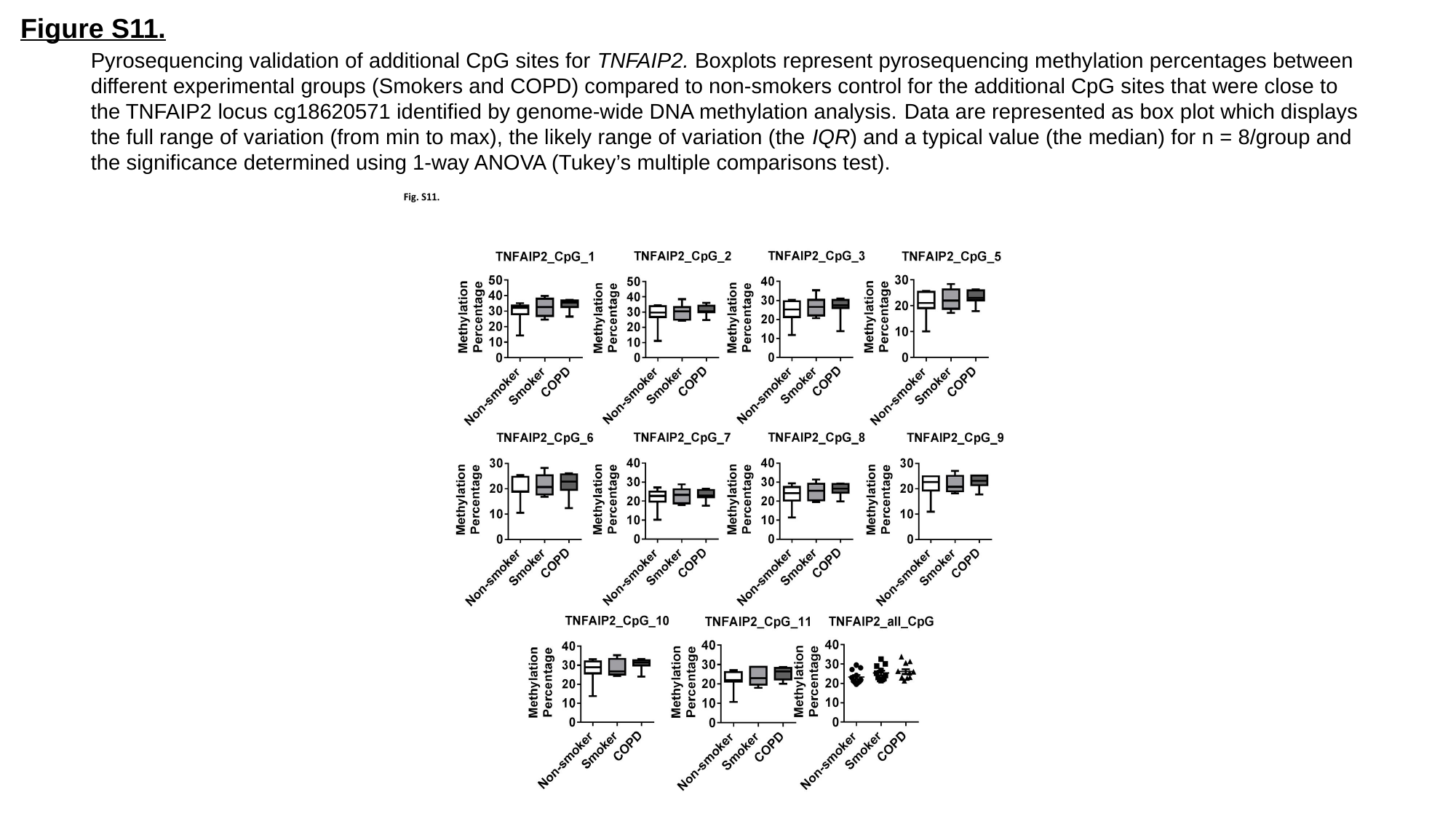

Figure S11.
Pyrosequencing validation of additional CpG sites for TNFAIP2. Boxplots represent pyrosequencing methylation percentages between different experimental groups (Smokers and COPD) compared to non-smokers control for the additional CpG sites that were close to the TNFAIP2 locus cg18620571 identified by genome-wide DNA methylation analysis. Data are represented as box plot which displays the full range of variation (from min to max), the likely range of variation (the IQR) and a typical value (the median) for n = 8/group and the significance determined using 1-way ANOVA (Tukey’s multiple comparisons test).

## Slide 12
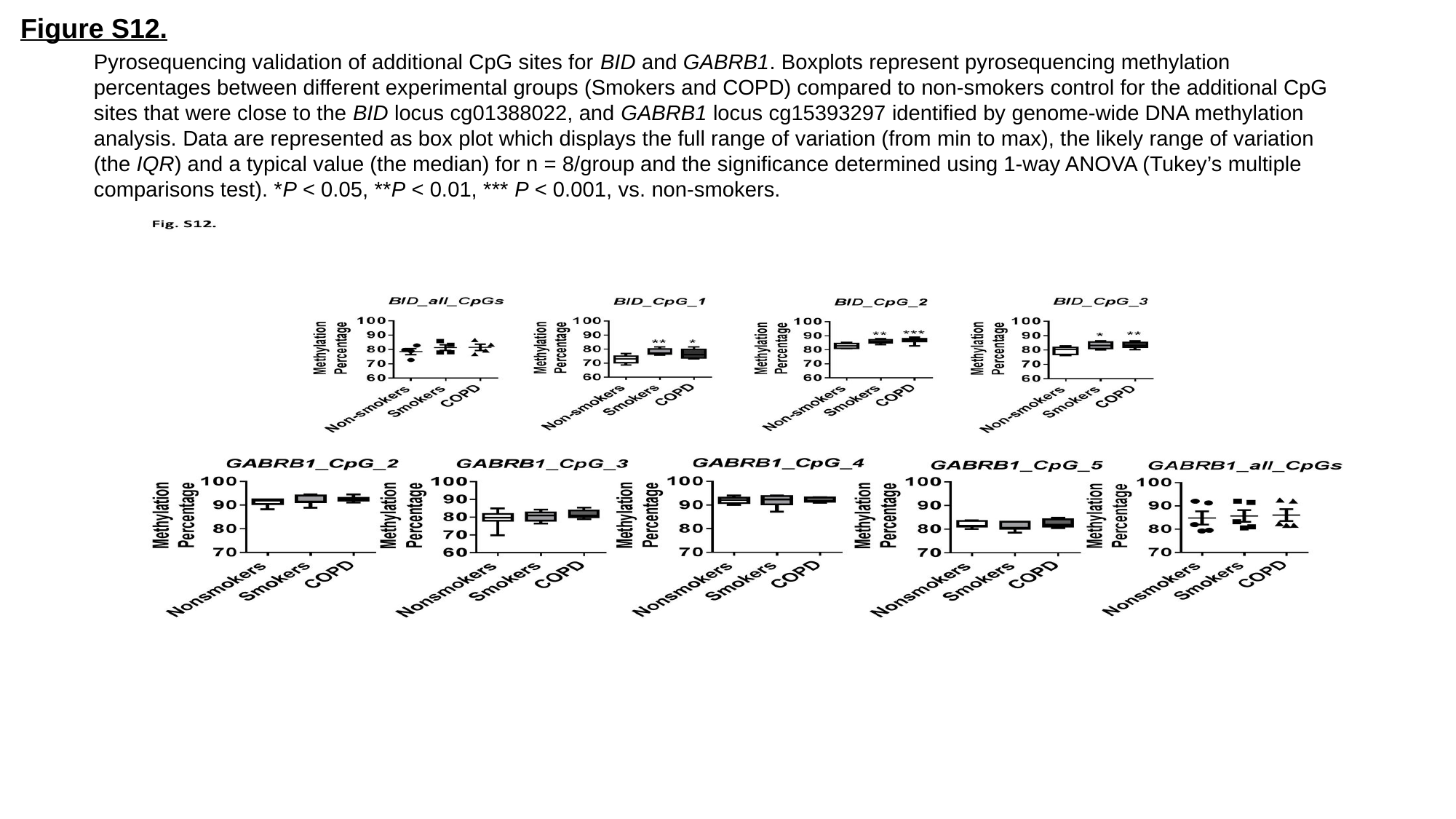

Figure S12.
Pyrosequencing validation of additional CpG sites for BID and GABRB1. Boxplots represent pyrosequencing methylation percentages between different experimental groups (Smokers and COPD) compared to non-smokers control for the additional CpG sites that were close to the BID locus cg01388022, and GABRB1 locus cg15393297 identified by genome-wide DNA methylation analysis. Data are represented as box plot which displays the full range of variation (from min to max), the likely range of variation (the IQR) and a typical value (the median) for n = 8/group and the significance determined using 1-way ANOVA (Tukey’s multiple comparisons test). *P < 0.05, **P < 0.01, *** P < 0.001, vs. non-smokers.

## Slide 13
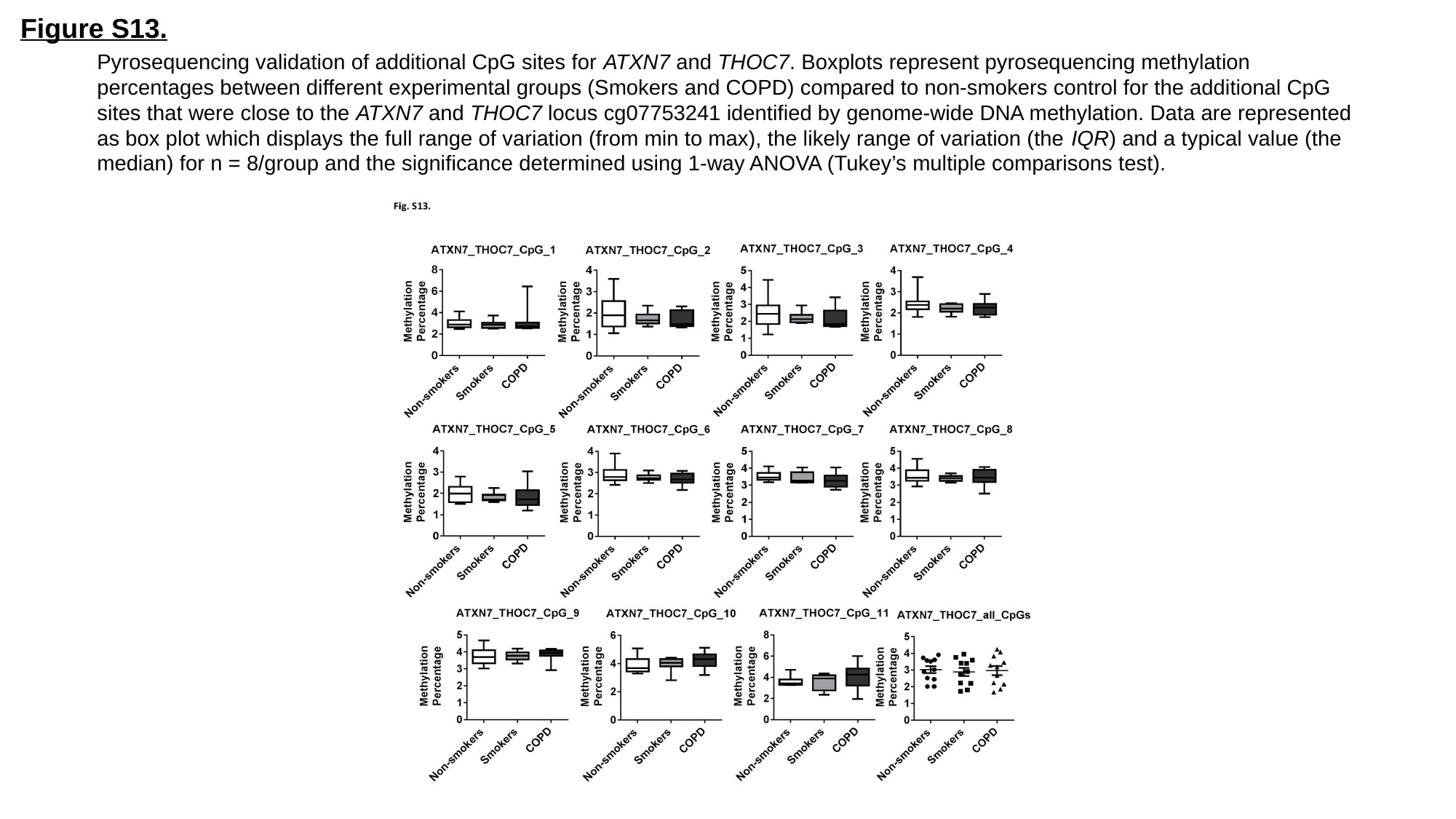

Figure S13.
Pyrosequencing validation of additional CpG sites for ATXN7 and THOC7. Boxplots represent pyrosequencing methylation percentages between different experimental groups (Smokers and COPD) compared to non-smokers control for the additional CpG sites that were close to the ATXN7 and THOC7 locus cg07753241 identified by genome-wide DNA methylation. Data are represented as box plot which displays the full range of variation (from min to max), the likely range of variation (the IQR) and a typical value (the median) for n = 8/group and the significance determined using 1-way ANOVA (Tukey’s multiple comparisons test).
